# Integrated immunogenomic analyses of high-grade serous ovarian cancer reveal vulnerability to combination immunotherapy

**DOI:** 10.3389/fimmu.2024.1489235

**Published:** 2024-11-28

**Authors:** Raphael Gronauer, Leonie Madersbacher, Pablo Monfort-Lanzas, Gabriel Floriani, Susanne Sprung, Alain Gustave Zeimet, Christian Marth, Heidelinde Fiegl, Hubert Hackl

**Affiliations:** ^1^ Institute of Bioinformatics, Biocenter, Medical University of Innsbruck, Innsbruck, Austria; ^2^ Institute of Medical Biochemistry, Biocenter, Medical University of Innsbruck, Innsbruck, Austria; ^3^ Institute of Pathology, Innpath GmbH, Innsbruck, Austria; ^4^ Department of Obstetrics and Gynecology, Medical University of Innsbruck, Innsbruck, Austria

**Keywords:** high-grade serous ovarian cancer, BRCAness, PARP inhibitor, immunotherapy, tumor-associated macrophages, precision oncology, tumor immune microenvironment

## Abstract

**Background:**

The efficacy of immunotherapies in high-grade serous ovarian cancer (HGSOC) is limited, but clinical trials investigating the potential of combination immunotherapy including poly-ADP-ribose polymerase inhibitors (PARPis) are ongoing. Homologous recombination repair deficiency or BRCAness and the composition of the tumor microenvironment appear to play a critical role in determining the therapeutic response.

**Methods:**

We conducted comprehensive immunogenomic analyses of HGSOC using data from several patient cohorts. Machine learning methods were used to develop a classification model for BRCAness from gene expression data. Integrated analysis of bulk and single-cell RNA sequencing data was used to delineate the tumor immune microenvironment and was validated by immunohistochemistry. The impact of PARPi and BRCA1 mutations on the activation of immune-related pathways was studied using ovarian cancer cell lines, RNA sequencing, and immunofluorescence analysis.

**Results:**

We identified a 24-gene signature that predicts BRCAness. Comprehensive immunogenomic analyses across patient cohorts identified samples with BRCAness and high immune infiltration. Further characterization of these samples revealed increased infiltration of immunosuppressive cells, including tumor-associated macrophages expressing *TREM2*, *C1QA*, and *LILRB4*, as specified by single-cell RNA sequencing data and gene expression analysis of samples from patients receiving combination therapy with PARPi and anti-PD-1. Our findings show also that genomic instability and PARPi activated the cGAS-STING signaling pathway *in vitro* and the downstream innate immune response in a similar manner to HGSOC patients with BRCAness status. Finally, we have developed a web application (https://ovrseq.icbi.at) and an associated R package OvRSeq, which allow for comprehensive characterization of ovarian cancer patient samples and assessment of a vulnerability score that enables stratification of patients to predict response to the combination immunotherapy.

**Conclusions:**

Genomic instability in HGSOC affects the tumor immune environment, and TAMs play a crucial role in modulating the immune response. Based on various datasets, we have developed a diagnostic application that uses RNA sequencing data not only to comprehensively characterize HGSOC but also to predict vulnerability and response to combination immunotherapy.

## 1 Introduction

Despite newer therapeutic concepts, ovarian cancer, particularly high-grade serous ovarian cancer, is still the deadliest gynecologic malignancy, with 13,270 expected deaths in 2023 in the U.S (1). While immunotherapy, such as immune checkpoint inhibition monotherapy (e.g., antibodies against PD-1 or PD-L1), has dramatically changed the therapeutic concepts of different cancer types, especially those with mismatch repair deficiency (2), the benefit for ovarian cancer patients with an objective response rate of approximately 10% was found to be rather modest (3–6). However, poly-ADP-ribose polymerase inhibitors (PARPis) and antiangiogenic therapy have improved the survival outcomes of ovarian cancer patients beyond standard care, namely, debulking surgery and platinum-based therapy (7). Furthermore, a number of clinical trials of combination therapies, including immune checkpoint blockade, are underway (8–12). Whereas the recent primary analysis of the double-blind placebo-controlled ENGOT-Ov41/GEICO 69-O/ANITA phase III trial showed that the addition of the anti-PD-L1 antibody (atezolizumab) did not significantly improve the clinical outcome (12), early analysis of the MEDIOLA phase II study adding the PD-L1 inhibitor (durvalumab) and the angiogenesis inhibitor (bevacizumab) to a PARPi (olaparib) was promising, with an objective response rate >90% for a specific patient group with platinum-sensitive relapsed ovarian cancer harboring germline *BRCA* mutations (11).

PARP is involved in DNA damage and repair, binds to single-strand DNA breaks, and performs posttranslational modifications of histones and DNA-associated proteins by poly-ADP-ribosylation, also known as parylation. PARP inhibitors trap PARP and stall the replication fork, which can subsequently cause DSBs. PARP inhibition is synthetic lethal with deleterious *BRCA1* and *BRCA2* mutations because homologous recombination repair (HRR) cannot restore these double-strand breaks, introducing genome instability by nonhomologous end joining or leading to tumor cell death (13). In high-grade serous ovarian cancer, approximately 14% harbor a germline and 6% a somatic mutation in the *BRCA1* or *BRCA2* gene, and approximately 50% are HRR deficient (HRD), indicating that they are favorable for PARPi therapy (14, 15). Sequencing approaches enable researchers to detect mutations in other genes involved in HRR. However, the concept of HRD or BRCAness goes beyond, as it encompasses instabilities and genomic scars, including large-scale transitions, loss of heterozygosity, telomeric allelic imbalance and specific mutational processes with uneven base substitution patterns (mutational signature 3). Several diagnostic assays from commercial providers for the detection of HRD have already been approved (16). However, further efforts are undertaken to identify various biomarkers based on different modalities, such as gene expression or methylation, in the context of different cancer types (17–21). Deleterious *BRCA1* mutations and/or PARP inhibition can trigger an immune response at least in part through the cGAS-STING pathway (22–26), suggesting advantages for combined immunotherapies. However, biomarkers or phenotypes to predict the response to therapies, including PARPis and immune checkpoint blockers, are lacking.

In this study, we conducted comprehensive immunogenomic analyses of HGSOC using data from multiple patient cohorts. Integrated gene expression analysis and machine learning on bulk and single-cell RNA sequencing data enabled the 1) development of a 24-gene expression classification model for BRCAness, 2) stratification of patient samples with BRCAness and high immune infiltration, whereby tumor-associated macrophages (TAMs) proved to be an important suppressive component, 3) identification of the activation of immune-related pathways such as the cGAS-STING or JAK-STAT pathway and downstream signaling by PARPi and BRCA1 mutation (BRCAness), and 4) the development of a diagnostic application from RNA sequencing data to comprehensively characterize HGSOC samples and predict vulnerability and response to combination immunotherapy.

## 2 Methods

### 2.1 Patient cohorts and datasets

The analysis workflow and used datasets from various cohorts are summarized in **Supplementary Figure S1**. Patient characteristics for the TCGA-OV cohort (n=226) (15) and the new HGSOC cohort from the Medical University in Innsbruck (MUI) (n=60) are listed in **Supplementary Tables S1** and **S2**. RNA sequencing data and clinical data for the validation cohort (Medical University of Innsbruck; MUI) were deposited at https://doi.org/10.5281/zenodo.10251467. Controlled access data for whole exome sequencing and RNA sequencing data for the TCGA-OV cohort were obtained through dbGaP access permission (phs000178). Processed data (including methylation beta values) and clinical data were downloaded from Firebrowse (firebrowse.org, BROAD Institute). Additional clinical data were retrieved from the supplementary data of another resource (27). Raw RNA sequencing data and clinical annotations for the ICON7 cohort (28) were downloaded from the EGA archive (EGAS00001003487). Single-cell RNA-seq data (29) were downloaded from the Gene Expression Omnibus (GEO) (GSE180661) as an annotated count matrix (anndata-object) in h5ad-format. Data files from the TOPACIO clinical trial (9) were retrieved from Synapse (https://doi.org/10.7303/syn21569629). Data from the Clinical Proteomic Tumor Analysis Consortium ovarian cancer cohort (CPTAC-OV) (30) were downloaded from (https://proteomics.cancer.gov) (n=71). Data from RNA sequencing analysis of OVCAR 3 and UWB1.289 cancer cell lines performed in this study were deposited in GEO (GSE237361). Only complete data sets were used, and observations (rows) with missing values were deleted before specific analyses were performed.

### 2.2 Cell line experiments

Two epithelial ovarian carcinoma cell lines, UWB1.289 harboring a deleterious BRCA1 and OVCAR3 with intact BRCA1, were obtained from ATCC. OVCAR3 cells were grown in RPMI 1640 with 0.01 mg/ml bovine insulin and 20% FBS, whereas UWB1.289 cells were grown in a mixture of 48.5% MEGM Bullet Kit medium (Lonza) and 48.5% RPMI 1640 with 3% FBS. Viability assays were used to determine the IC50 for olaparib. Both cell lines were treated with olaparib or DMSO for 96 hours in four replicates. Treated and untreated UWB1.289 and OVCAR3 cells were stained with indirect immunofluorescent antibodies to detect γH2AX as an indicator of double-strand breaks. To determine activated STING signaling, double-stranded DNA and its presence in the cytosol, cGAS, STING, and phosphorylated STING were detected. The antibodies used are listed in **Supplementary Table S3**.

### 2.3 Immunohistochemistry analyses

Slices of 10 selected tumor blocks were subjected to immunohistochemistry analyses performed on the BenchMark ULTRA automated staining device (Ventana, Oro Valley, AZ/Roche, Vienna, Austria). The examined markers were CD163 for macrophages and CD8, PD-1, CD4, and FOXP3 for T cells. Furthermore, the markers γH2AX and STING were analyzed. All antibodies used are listed in **Supplementary Table S4**.

### 2.4 RNA sequencing analyses

RNA from cancer cell line samples was isolated from 2x10^6^ cells each using the RNeasy Mini Kit (Qiagen) according to the manufacturer’s protocols. RNA quantity and quality were assessed using NanoDrop™ 2000c and Bioanalyzer 2100 with Agilent 6000 Nano Kit and cDNA libraries were generated using the QuantSeq 3’ mRNA-Seq Library Prep Kit (Lexogen) according to the manufacturer’s instructions. Paired-end sequencing (150 bp) was performed on a NovaSeq 6000 sequencing device at GENEWIZ/Azenta. RNA isolation from 60 fresh frozen tumor samples from the MUI HGSOC validation cohort was conducted in a similar manner resulting in sufficient quality (RIN factors from 6.4 to 9.9), and sequencing was performed at Novogene (Cambridge, UK) for paired end sequencing (PE150) on an Illumina NovaSeq 6000 sequencing device using TrueSeq (Illumina) strand-specific total RNA libraries.

### 2.5 RNA sequencing data analyses

Raw reads were quality checked using FastQC. Reads were mapped to the human reference genome version hg38 (GRch38) using STAR (version 2.7.1) in 2pass mode (31). Gene level expression quantification was performed with featureCounts (version 2.0.0) using GENCODE annotations (v36). Raw counts were normalized using TPM (transcripts per million). RNA sequencing raw data from the MUI HGSOC cohort and the ICON7 cohort were analyzed in the same way. For sequencing data of the cell lines, single-end reads were processed by trimming adapter and low-quality sequences using BBDuk with the parameters specified by Lexogen. The trimmed reads were mapped to the human reference genome version hg38 (GRch38) using STAR (version 2.7.9a) in 2-pass mode. Gene level expression quantification was performed with featureCounts (version 2.0.0) and GENCODE annotations (v38).

### 2.6 Whole exome sequencing analyses and variant calling

Raw exome sequencing reads in fastq format were quality checked using FastQC. Reads of paired tumor and normal samples were mapped against the human reference genome version hg38 (GRch38) using BWA. For the detection of germline variants the HaplotypeCaller was used. To assess somatic variants in the tumor samples, four different variant callers, Mutect2 (32), SomaticSniper (33), Varscan2 (34), and Strelka2 (35) were used. If a variant was called by two of four variant callers and the variant allele frequency was ≥ 0.05 in the tumor sample and <0.05 in the normal sample, the variant passed filtering. Variants were annotated using VEP (36) with the ClinVar extension. Only pathogenic (class V) and likely pathogenic (class IV) variants were considered to affect the function of HRR genes such as *BRCA1* or *BRCA2*. Tumor mutational burden was calculated based on the number of nonsynonymous single nucleotide variants per megabase for each tumor sample. For neoantigen prediction, from somatic mutation derived peptide sequences with lengths between 8-11 amino acids - taking phasing into account - were generated and tested for the respective HLA alleles with NetMHCpan-4.0 (37), whereby %rank<2 was considered a weak binder and %rank<0.5 was considered a strong binder. Dissimilarity to the normal human proteome (hg38) was identified by the *antigen.garnish* package. Neoantigen load was calculated for each tumor based on predicted weak and strong binding neoantigens – irrespective of their peptide length and taking all HLA alleles into account – per megabase.

### 2.7 Functional analysis of gene expression and the tumor immune environment

Differential gene expression analysis was conducted using the R package DESeq2 (38). P values were adjusted for multiple testing based on the false discovery rate (FDR) according to the Benjamini−Hochberg method. Genes with more than a twofold change at an FDR<0.1 and average expression across all samples (baseMean>10) were considered significantly differentially expressed. To identify functional annotation and affected biological processes, log2-fold change preranked gene set enrichment analyses (GSEA) (39) using hallmark and selected immune-related gene sets from MSigDB were performed. ClueGO was used to build a network and group significantly overrepresented pathways, which share genes (40). The STRING database (https://string-db.org/) was used to identify an interaction network within the differentially expressed genes, and subnetworks were found by MCL clustering with inflation parameter=3. Footprint analyses of response genes of perturbed cancer signaling pathways were performed using PROGENy (41). To assess tumor infiltration of immune cells quanTIseq (42) using the immunedeconv R package was applied to bulk RNA sequencing data. To characterize the immune-related processes, well-described immune signatures, such as T-cell inflammation, IFN gamma signature, cytolytic activity, cytotoxic T lymphocyte function, and T-cell exhaustion (**Supplementary Table S5**), were analyzed. Based on log2(TPM+1) normalized expression data, single sample gene set enrichment using GSVA (43) was performed for signatures with more than 10 genes or otherwise average expression was calculated. The tumor-immune phenotype (infiltrated, excluded, desert) was determined based on a previously developed classification model based on 157 genes using digital pathology describing the presence and position of CD8+ T cells relative to the center or margin of the tumor (28) and a random forest model was used to characterize samples from the TCGA and the MUI cohorts. To classify ovarian cancer samples into molecular subtypes, the consensusOV R package (44) was used. The immunophenoscore (IPS) was determined as described previously (45).

### 2.8 Determination and classification of BRCAness

BRCAness was determined based on HRD scores (46), mutational signature 3 (47), mutations in homologous recombination repair pathway genes and methylation of promoter regions of BRCA1. All HGSOC samples of the TCGA cohort for which paired tumor and normal exome sequencing and matched RNA sequencing data were available (n=226) were used. Samples were classified with a BRCAness phenotype when they had either a deleterious mutation in the homologous recombination pathway, an ovarian cancer-specific HRD score of ≥ 63 (48), a mutational signature 3 ratio > 0.25 or a methylation level beta value >0.7 of the BRCA1 promoter. HRD scores were calculated as the unweighted sum of the three genomic scar values, loss of heterozygosity (LOH) (49), telomeric allelic imbalance (TAI) (50), and large-scale state transitions (LST) (51). To compute the genomic scar values, scarHRD (52) was used on genome segmentation files generated with Sequenza (53). The mutational signature 3 score was computed using MutationalPatterns (54) and we calculated the ratio between mutational signature 3 supporting mutations and all detected mutations. To classify BRCAness, genes expressed in at least one ovarian cancer cell were identified using single-cell RNAseq data. Normalized gene expression values (log2 (TPM+1)) of these genes in the TCGA dataset were then subjected to recursive feature elimination in a balanced design with three different machine learning models (random forest, AdaBoost and gradient boosting) to identify the 50 most important features for each model. Since ensemble methods and random subsampling (bootstrap) were included, we did not use nested cross-validation to avoid overfitting. Genes that were among the top 50 in at least two of the three models (24 genes) were then used subsequently to train a random forest classification model to discriminate between BRCAness and noBRCAness samples based on gene expression data. The performance of the classifier was evaluated by analysis of the receiver operating characteristic curve with 10-fold cross-validation. The area under curve (AUC) was used as a performance measure. A cutoff for BRCAness (P>0.5266) was selected using the Youden index. Furthermore, the classifier was tested in 29 patients of the independent validation cohort (MUI) with HRD information based on SNP arrays and further validation using Myriad MyChoice CDx. Samplewise BRCA classification in single-cell RNA sequencing data from 29 patients was performed with an optimized cutoff (P>0.45) and based on the majority of classified tumor cells. A method to detect mutational signature 3 (Sig3), termed SigMA, from clinical panel sequencing data have been previously developed and associated with HRD (55). To further compare our BRCAness classifier, which is based on a BRCAness definition more completed as compared to the other methods described in literature such as the SigMA score, using available RNA sequencing and mutation data from the Clinical Proteomic Tumor Analysis Consortium ovarian cancer (CPTAC-OV) cohort (30).

### 2.9 Single-cell RNA sequencing analysis

All analyses of single-cell data were performed in Python using scanpy (56) and scvi-tools (57). Since the samples were sequenced separately for sorted CD45+ and CD45- cells, the raw read counts were integrated using scvi-tools with a batch effect correction. Counts were normalized to counts per million (CPM) and log2 transformed, adding a pseudocount of 1. Quality metrics were determined using scanpy and filtered for genes that are expressed in at least one cell. The dataset was filtered for samples from the primary tumor (adnexal tumor tissue). Principal component analysis and nearest neighbor analyses were calculated with default settings, and clustering was performed with the Leiden algorithm. Super cell types were annotated as previously defined. Subtypes of T-cell and myeloid cell clusters were assigned based on the expression of marker genes using published marker genes for different cell types and the PanglaoDB (58). Differentially expressed genes between clusters were calculated using the Wilcoxon ranked sum test. For visualization, we used uniform manifold approximation and projection (UMAP) dimensional reduction. Gene expression between cell types was compared by heatmaps, violin plots, and bubble plots. Distribution of cell fractions for each sample or combined for BRCAness and noBRCAness group were compared by stacked barplots and two-sided Wilcoxon rank sum test. Pseudobulk analyses and DESeq2 analyses was performed for selected immune response markers to test effect of BRCAness *versus* noBRCA samples on the immune response. To assess ligand−receptor interactions between cancer cells and cells from the TME, CellPhoneDB (59) analysis was used.

### 2.10 Gene expression analysis

Gene expression analysis in the TOPACIO cohort was performed on the NanoString platform. We used the nSolver software from NanoString (Seattle, US) to obtain normalized data. Differential expression analysis was performed using the R package limma (60), and genes with p<0.05 were considered differentially expressed.

### 2.11 Vulnerability score and maps

Vulnerability maps consist of three variables: the vulnerability score, the BRCAness score and the cytolytic activity (CYT) to *C1QA* ratio. For the BRCAness score, the prediction probability from the random forest classifier was used. The CYT to *C1QA* ratio was calculated from the log2 (TPM+1) values of *GZMB*, *PRF1*, and *C1QA* (Equation 1).


(1) CYT to C1QA ratio=0.5 * (GZMB+PRF1)/C1QA

The CYT to *C1QA* ratio was transformed to values between 0 and 1 using a sigmoid function with softmax transformation and parameters derived from the TCGA cohort and termed C2C (Equation 2).


(2) $$C2C=1/\left(1+e^{-\frac{CYT\;to\;C1QA\;ratio-0.301}{0.0433}}\right)$$

The vulnerability score was defined as the weighted sum of BRCAness probability and C2C (Equation 3), whereby the weights were identified using a logistic regression model on the CYT to *C1QA* ratio using log2 intensity expression values and SigMA status (mutational signature 3) as proxy for BRCAness from the TOPACIO cohort and the treatment response as a binary dependent variable.


(3) Vulnerability score=2.597 * BRCAness probability+1.166 * C2C

For visualization of the vulnerability map, a two-dimensional map was created with C2C as one coordinate, BRCA probability as the other coordinate, and the color-coded vulnerability score.

### 2.12 Statistical analysis

Survival analyses were performed for both HGSOC cohorts (TCGA, MUI) for selected genes, immune parameters, or immune cell fractions by dichotomization of patients based on the median or maximum log-rank statistics using the R package survival. For the TCGA cohort, overall survival (OS) and progression free survival (PFS) survival status were derived from a clinical data resource for TCGA (27) and for the cohort from Medical University Innsbruck from the clinical data as provided by the Department of Obstetrics and Gynecology. Univariate and multivariable Cox regression analyses taking clinical parameters into account (age, FIGO stage, residual tumor) were performed, Kaplan-Meier survival curves were generated, and compared by log rank test. To determine the association between continuous or binary variables, point biserial correlation analysis was used. For the correlation between binary variables, the Phi coefficient and chi-square test were used, and for the correlation between continuous variables, Pearson’s correlation coefficient was used. To compare parameters between two groups, the Wilcoxon rank-sum test was used. For multiple group comparisons the non-parametric Kruskal-Wallis test followed by pairwise two-tailed Dunn posthoc tests with p-value adjustment based on the false discovery rate (FDR) were used. Where indicated, p values were adjusted for multiple testing based on the FDR according to the Benjamini−Hochberg method. P<0.05 or FDR<0.1 were considered significant.

## 3 Results

We performed immunogenomic characterization and multimodal integrative analyses of data from several patient cohorts, including the TCGA cohort (n=226), the MSK cohort (n=29), the ICON7 cohort (n=327), the CPTAC cohort (n=71), patients from the TOPACIO study receiving combination immunotherapy (n=22), and a new MUI cohort for validation (n=60). The used data modalities and complete analysis workflow is outlined in **Supplementary Figure S1**.

### 3.1 A 24-gene signature predicts BRCAness in HGSOC patients

**Figure 1:**
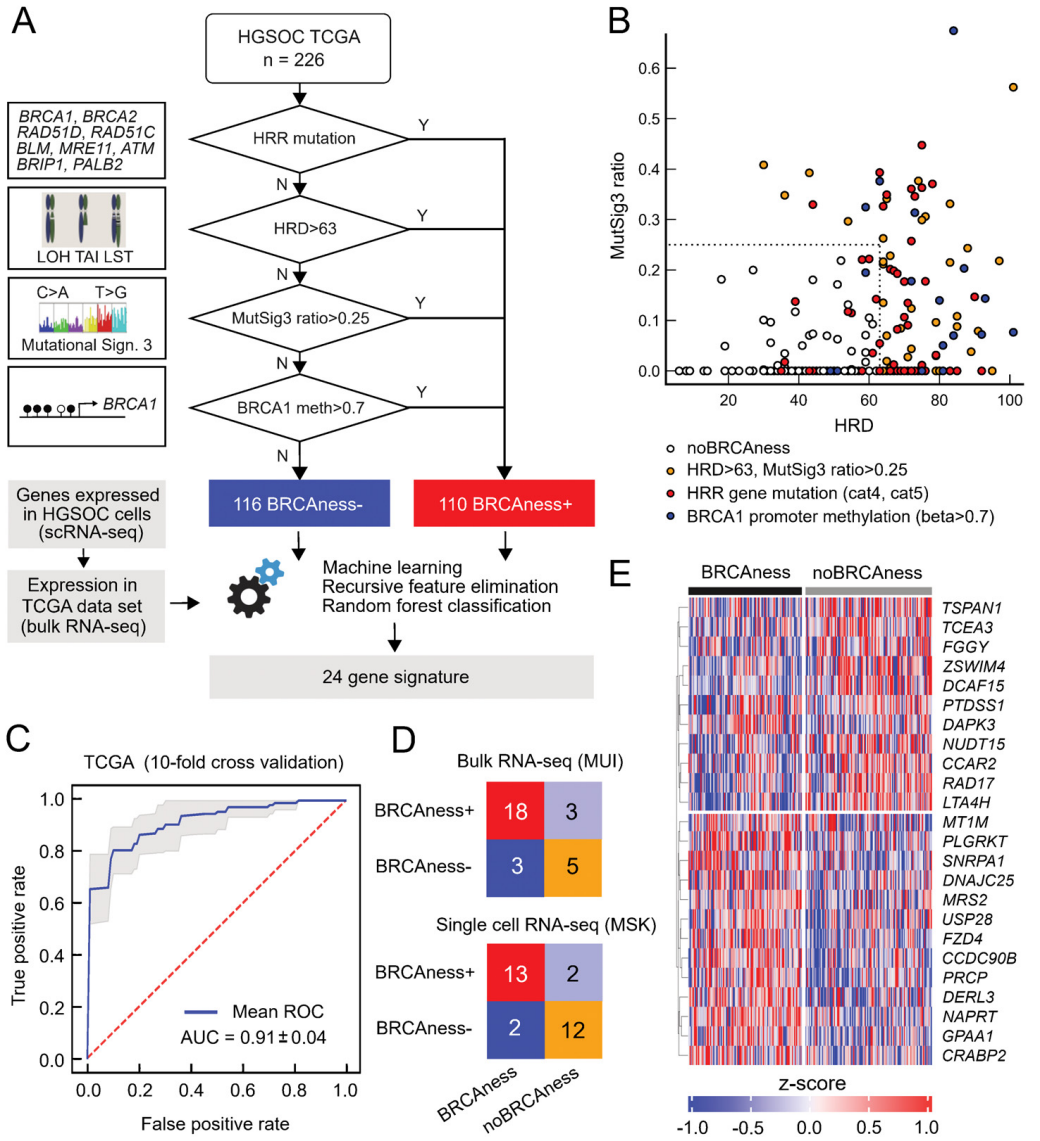
BRCAness classification based on the expression of 24 genes. **(A)** Determination of BRCAness in the TCGA-OV cohort and the development of a gene expression-based BRCAness classifier. **(B)** Different BRCAness parameters in the TCGA cohort compared between the HRD score and the mutation signature 3 ratio. Samples with mutated homologous recombination repair pathway genes are marked in red, BRCA1/2 promoter methylation in blue and samples with an HRD score > 63 and/or a signature 3 ratio > 0.25 but no mutation or BRCA1/2 promoter methylation are marked in yellow. Samples without BRCAness are marked in white. **(C)** Mean ROC curve with 10-fold cross-validation of the classifier tested on the TCGA dataset. **(D)** Confusion matrices with correctly and incorrectly classified instances when the classifier was tested in independent test cohorts of single-cell RNA sequencing and bulk RNA sequencing data. **(E)** Z scores of log2(TPM+1) normalized expression of the 24 genes of the BRCAness signature in the TCGA cohort as a heatmap clustered by BRCAness and non-BRCAness samples.

Because the response to platinum-based chemotherapies or therapy with PARP inhibitors in ovarian cancer is not limited to patients with tumors harboring *BRCA1* or *BRCA2* mutations, we expanded the group of patients by using a genomic characterization termed BRCAness, which has very much in common with homologous recombination repair deficiency (HRD) status (61). BRCAness status includes mutations of genes in the homologous recombination DNA repair pathway (HRR), genomic scars, loss of heterozygosity, telomeric allelic imbalance, or large-scale transitions, mutational signature 3, or promoter methylation of the *BRCA1* or *BRCA2* gene. We assessed these parameters based on whole exome sequencing data and methylation data from the TCGA OV cohort (**Figure 1A**). Very few patients harboring HRR mutations or *BRCA1* promoter methylation fell below the combination of the HRD cutoff (HRD>63) and the MutSig3 ratio cutoff (0.25), indicating a reasonable selection of the cutoff values (**Figure 1B**). To identify BRCAness solely based on gene expression data, we developed a machine learning classifier that can discriminate between BRCAness and non-BRCAness samples using bulk and single-cell RNA sequencing data (**Figure 1A**). Recursive feature elimination based on multiple models resulted in a BRCAness gene expression signature with 24 genes, which was used to train a random forest model discriminating between BRCAness and noBRCAness. The receiver operating characteristics with 10-fold cross-validation on the training dataset showed an area under the curve (AUC) of 0.91 ± 0.04 (**Figure 1C**). To validate our BRCAness classifier we tested its performance in two independent ovarian cancer cohorts. We could classify BRCAness in a new HGSOC cohort from the Medical University Innsbruck (MUI) (n=60) based on RNA sequencing data with an accuracy of 0.79, an F1-score of 0.86, and a positive prediction value of 0.86 (**Figure 1D**). Furthermore, we demonstrated that in addition to classifying bulk RNAseq samples the classifier is also capable of classifying samples from single cell RNA-seq data at the sample level in the HGSOC MSK cohort (n=29) with an accuracy of 0.86, an F1 score of 0.87 and a positive prediction value of 0.87 (**Figure 1D**).

There was also good agreement with a recently defined gene expression-based HRDness signature including 173 up- and 76 downregulated genes (62) using a single sample gene set enrichment derived score in the TCGA cohort as well as the MUI validation cohort with Spearman’s rank correlation of ρ=0.72 (P<0.001) and ρ=0.63 (P<0.001), respectively (**Supplementary Figures S2**, **S3**). Interestingly, six genes from our 24-gene signature (**Figure 1E**) to classify BRCAness (*CCDC90B*, *CRABP2*, *FZD4*, *GPAA1*, *PRCP*, *SNRP1*) were also among the upregulated and two genes (*RAD17*, *LTA4H*) among the downregulated genes. The 24-gene BRCAness signature was further compared to the SigMA score (mutational signature 3) of the CPTAC-OV cohort (n=71). Although our BRCAness signature is based on a more complete BRCAness definition than the mutational signature, a significant correlation was observed with a Spearman’s rank correlation of ρ=0.43 (P<0.001) (**Supplementary Figure S4**). We used this BRCAness classification for all remaining analyses.

In summary, we developed a 24-gene-based BRCAness model validated in several single-cell and bulk RNAseq datasets with reasonable classification performance.

### 3.2 Genome instability is associated with immune-related processes

**Figure 2:**
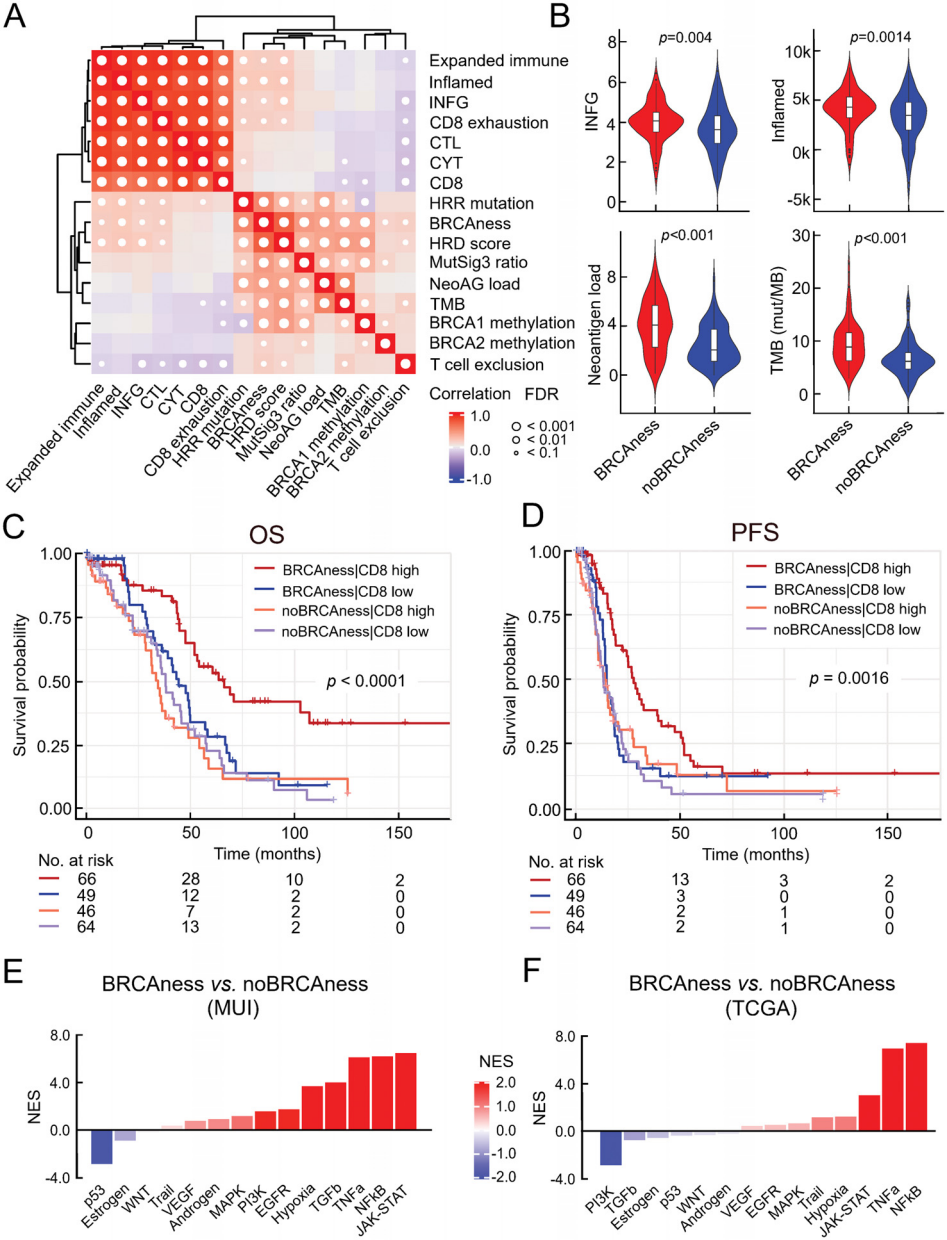
Association between BRCAness and immune parameters. **(A)** Results of correlation analysis of selected immune signatures and BRCAness parameters in the TCGA-HGSOC cohort (CYT, cytolytic activity; CTL, cytotoxic T lymphocytes; IFNG, interferon gamma signature; HRR mutations, mutations in the homologous recombination repair pathway; NeoAG load, neoantigen load; TMB, tumor mutational burden); white dots indicate significance (FDR<0.1). **(B)** Direct comparison of selected immune parameters between BRCAness and noBRCAness samples with significant differences, Wilcoxon rank-sum test (FDR<0.1) in the TCGA cohort (n=226). **(C)** Kaplan−Meier curves according to overall survival (OS) and for 4 patient groups of the TCGA dataset (n=226) based on BRCAness information and median dichotomized estimated CD8 T cell fraction (quanTIseq): patients with 1) BRCAness and high estimated CD8 T cell fraction, 2) BRCAness and low estimated CD8 T cell fraction, 3) noBRCAness and high estimated CD8 T cell fraction, and 4) noBRCAness and high estimated CD8 T cell fraction (p-value is from logrank test). **(D)** Kaplan−Meier curves according to progression free survival (PFS) for the same groups of patients from the TCGA cohort (n=226) as in **(C)**. **(E, F)** Waterfall plot of normalized enrichment scores (NES) for the footprint analysis of immune-related pathways with PROGENy between BRCAness and non-BRCAness samples in the MUI (n=60) and TCGA (n=226) cohort.

To identify the relationship between genomic instability and the activation of the immune system, we performed correlation analyses between the BRCAness status and various immune-related signatures in the TCGA cohort (n=226). BRCAness could be significantly positively associated with the enrichment of immune-related signatures, such as those for IFNG response (rho=0.38, p=0.004) and T-cell inflamed tumor microenvironment (rho=0.46, p=0.0014), even to a larger extent with high tumor mutational burden (p<0.001) and high neoantigen load (p<0.001) (**Figures 2A, B**). However, compared to other cancer types with defective DNA mismatch repair the TMB or neoantigen load in ovarian cancer is rather low. Thus, increased immune activity is more indicative of deficient HRR. It is known that BRCAness is associated with longer overall survival, indicating that those patients are more responsive to platinum-based chemotherapy. In order to determine to which extend this could be explained by higher immune cell infiltration we estimated CD8+ T-cell infiltration from RNA sequencing data using quanTIseq and divided patients into 4 groups: BRCAness patients with high CD8 T cell fraction, BRCAness patients with low CD8 T cell fraction, noBRCAness patients with high CD8 T cell fraction, and noBRCAness patients with low CD8 T cell fraction. We observed a significant association with overall survival (p<0.0001; log rank test) (**Figure 2C**) and progression-free survival (p=0.0016; log rank test) (**Figure 2D**), with the BRCAness group with a high proportion of positive CD8 T cells being associated with the longest survival. Multivariable Cox regression analysis showed a significant effect of BRCAness *vs.* no BRCAness on overall survival (p=0.00062, HR=0.51with 95%-CI 0.35-0.75), while the impact of CD8 T cell was not significant (**Supplementary Table S6**) indicating a more pronounced role of the BRCAness status. Nevertheless, analyses of signaling pathways by downstream target expression using PROGENy indicated for the TCGA cohort (n=226) as well as the MUI validation cohort (n=60) that immune-related pathways, including TNFa, NFkB, and JAK-STAT, were activated in the BRCAness samples (**Figures 2E, F**). Using STRING analyses in the MUI cohort (n=60), we also identified a highly connected network including various chemokines and interleukins and their respective receptors (CCL7, CCL11, CXCL5, CXCL9, CXCL13, CCR2, CCR3, CCR4, CCR8, CXCR3, and IL6), which were significantly higher expressed in BRCAness tumors than in non-BRCAness tumors, indicating attraction and interaction with various immune cells (**Supplementary Figure S5**).

Essentially, we observed a correlation of BRCAness with various immune-related processes and, in particular, a group of patients with BRCAness and high CD8 T-cell proportion associated with longer survival times in HGSOC patients.

### 3.3 PARP inhibition activates the cGAS-STING pathway *in vitro*

**Figure 3:**
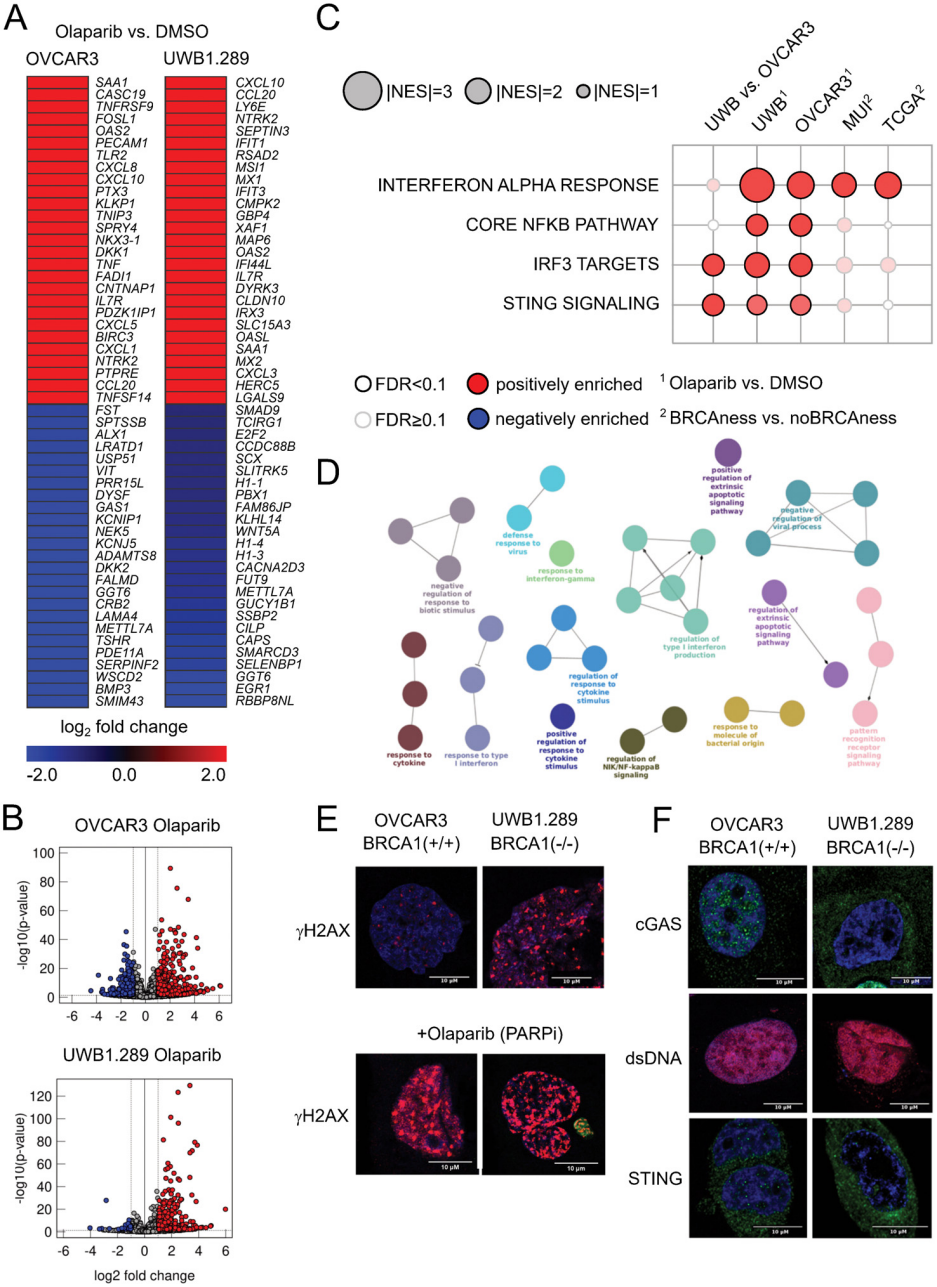
Results from cell line experiments with olaparib treatment **(A)** Top up- and downregulated genes for the cell lines OVCAR3 and UWB1.289 under olaparib treatment when compared to DMSO control. **(B)** General distribution of up- and downregulated genes after olaparib treatment compared to the DMSO control in both cell lines as volcano plots. Red indicates significantly upregulated genes (FDR<0.1, log2-fold change>1), and blue indicates significantly downregulated genes (FDR<0.1, log2-fold change<-1). **(C)** Normalized enrichment score of pathways associated with activation of the cGAS STING pathway in BRCA1 mutated (cell lines) and BRCAness samples (cohorts) as well as olaparib-treated cell lines. **(D)** ClueGO network indicating overrepresented biological processes in the olaparib-treated UWB1.289 cell line. **(E)** Immunofluorescence staining of the DNA damage marker γH2AX in OVCAR3 and UWB1.289 cell lines with and without olaparib treatment. Comparing the different response to PARPi treatment between BRCA1 mutation and wild type BRCA1 **(F)** Immunofluorescence staining of cGAS, double stranded DNA (dsDNA) and STING in the OVCAR3 and UWB1.289 cell line comparing the difference between BRCA1 mutation and wild type BRCA1.

To study the effect of PARPis on immune activation, we performed *in vitro* analyses. As tumor models, an ovarian cancer cell line with a proficient BRCA1 gene (OVCAR3) and a cell line with a mutation in the BRCA1 gene (UWB1.289) were used. We performed RNA sequencing analyses to identify differentially expressed genes between olaparib (PARPi)-treated and control (DMSO)-treated cell lines. Significantly upregulated genes (**Figures 3A, B**) indicate activation of various processes (**Figures 3C, D**), including pattern recognition receptor activation, response to cytokine signaling, interferon alpha response (type I), NFkB pathway, and cGAS-STING signaling. To further validate the results at the protein level, we performed immunofluorescence analyses indicating effects on γH2AX –a surrogate marker of DNA damage - by mutation in the BRCA1 gene and an even stronger effect by olaparib (PARPi) treatment (**Figure 3E**). Similarly, we observed a different activation of cGAS and STING – and indicating activation of the (innate) immune system – in the *BRCA1*-deficient versus the *BRCA1*-proficient cell model (**Figure 3F**). Furthermore, using gene set enrichment analyses, a significant interferon alpha response was also observed in BRCAness samples of both the TCGA cohort and the MUI validation cohort (**Figure 3C**).

In summary, we observed cGAS-STING activation by olaparib treatment *in vitro* and an interferon type I response as well as chemokine expression in HGSOC patient cohorts with BRCAness status.

### 3.4 BRCAness and immune subtype stratifies HGSOC patients

**Figure 4:**
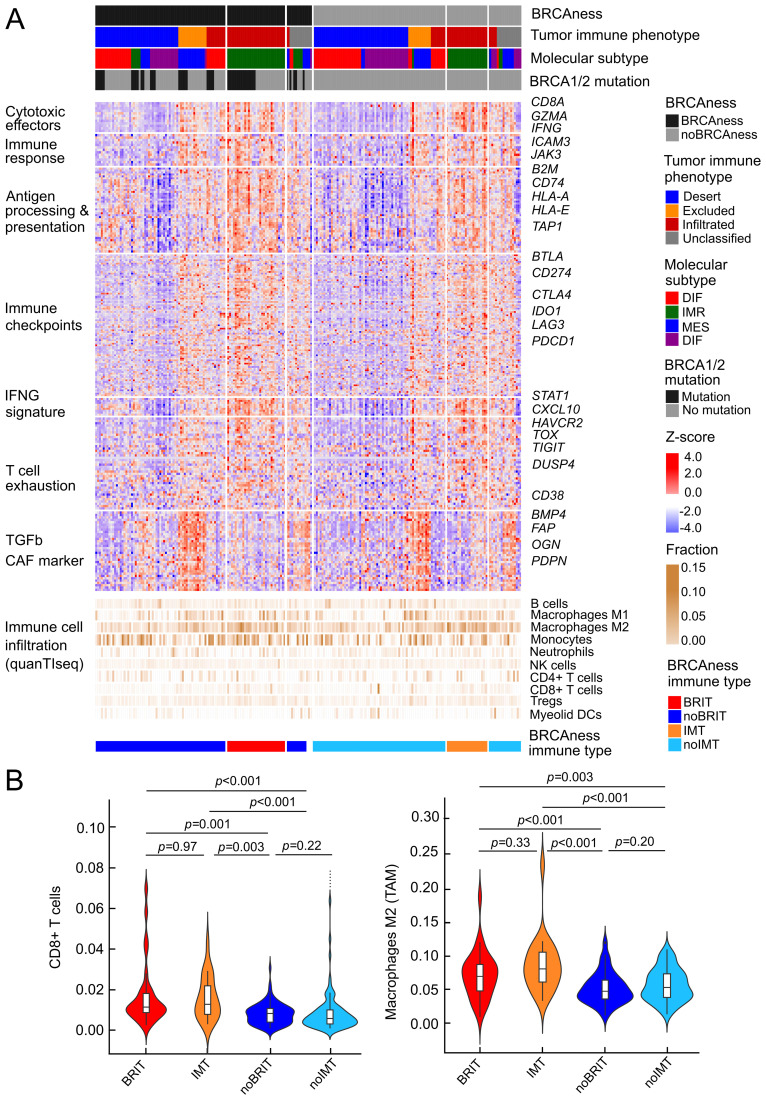
Profiles of immune parameters in the TCGA HGSOC cohort (n=226) **(A)** Heatmap of z scores of log2(TPM+1) expression of immune-related genes and fraction of tumor infiltrating immune cells assessed with quanTIseq and patient samples categorized by BRCAness, tumor-immune phenotype, molecular subtype and BRCA1/2 mutation. Furthermore, samples are stratified into four different tumor subtypes 1) BRCAness immune type samples (BRIT), which show an immunoreactive molecular subtype and an infiltrated tumor-immune phenotype, 2) noBRIT samples, which only have BRCAness but do not fulfill the other two requirements, 3) samples with an immune type (IMT) including an immunoreactive molecular subtype and an infiltrated tumor-immune phenotype but noBRCAness, and 4) remaining noIMT samples. **(B)** Distribution of estimated CD8 T cell fractions and estimated M2 macrophage fraction (from quanTIseq analyses) in the four different tumor subtypes. Benjamini-Hochberg adjusted p-values from pair-wise two-sided Dunn’s posthoc test are indicated.

We next focused on characterizing the presence of cytotoxic T lymphocytes and their spatial distribution in the tumor, following a recent approach in which digital pathology could be linked to gene expression (28). With the reported list of 157 genes and using random forest analysis, we were able to divide the patients of the TCGA cohort into a group with infiltrated, excluded, or desert tumor-immune phenotypes. Interestingly, the excluded phenotype was associated with upregulation of TGFβ and high expression of markers for cancer-associated fibroblasts, such as FAP or PDPN, which could form a barrier to prevent T-cell infiltration (**Figure 4A**). The immunoreactive molecular subtype (IMR) is very relevant to delineate immunoreactivity because many of the immunity genes, including cytotoxic effectors, factors involved in antigen processing and presentation, or immune checkpoints, are highly expressed in this condition (**Figure 4A**, **Supplementary Table S7**). We have selected a group of patients with tumor BRCAness, an infiltrated tumor immune phenotype and an immunoreactive molecular subtype called BRCAness immune type (BRIT), which we expect to respond well to combination immunotherapy. When comparing the estimated immune cell infiltrates in these cancer samples with BRCAness cancers without immune type (noBRIT), we found that not only cytotoxic T lymphocytes such as CD8+ T cells were significantly more abundant (p=0.001) but also a number of suppressive immune cells (M2 macrophages (p<0.001), regulatory T cells (p=0.005), myeloid-derived suppressor cells; MDSCs (p<0.001) (**Figure 4B**, **Supplementary Figure S6**). This is in line with previous observations (63) and in order to identify an effect by BRCAness we additionally defined an immune type (IMT) with noBRCAness, infiltrated tumor immune phenotype (INF), and an immunoreactive molecular subtype (IMR). However no significant difference between BRIT and IMT as well as noBRIT and noIMT could be observed for the analyzed cell types (**Figure 4B**, **Supplementary Figure S6**) indicating a more pronounced effect by immune infiltration than by BRCAness. Furthermore, we did not observe a significant difference in overall survival between BRIT *versus* noBRIT patients (p=0.56, HR =0.81, 95% CI 0.42-1.60).

These observations underscore the importance of the suppressive immune environment and suggest that suppressive immune cells may be an important factor, which is why ovarian cancer patients have a limited response to immunotherapy.

### 3.5 Tumor-associated macrophages inform therapy response

**Figure 5:**
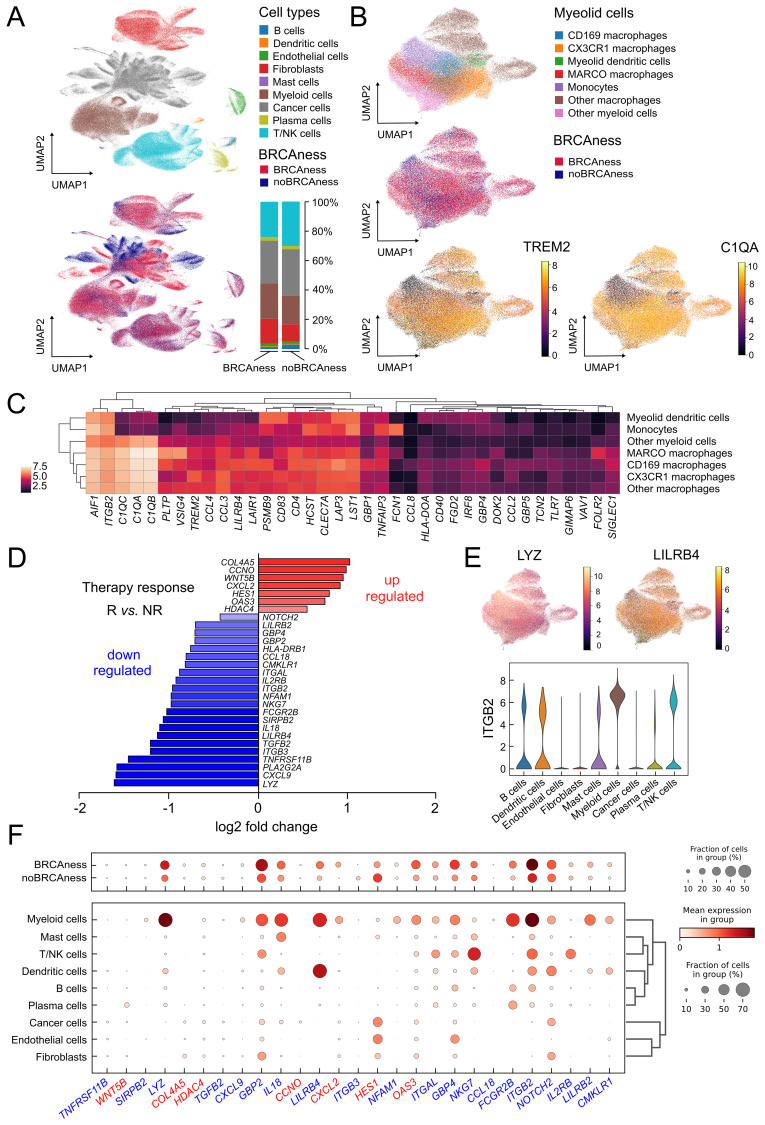
Single cell analysis of ovarian cancer adnexal samples from the MSK dataset (n=29) **(A)** UMAP showing the different cell types in of the ovarian cancer samples and which cells and cell types are associated with BRCAness samples. Distribution of major cell types in the BRCAness and noBRCAness are summarized as stacked bar plots. **(B)** UMAP plots of the myeloid cell compartment showing the association of macrophages with BRCAness cells and the expression of the macrophage marker gene C1QA and the TAM marker gene TREM2 especially in cell clusters associated with BRCAness. **(C)** Heatmap of expression of macrophage associated marker genes in the different cell types in the myeloid cell compartment. **(D)** Log_2_ fold changes of differentially expressed genes between responder [R] and non-responder [NR] to PARPi-immune checkpoint inhibition combination therapy (niraparib and pembrolizumab) from the TOPACIO clinical trial (n=22) (p<0.05) **(E)** Distribution of expression visualized by UMAP and violin plots indicating in which (myeloid) cell types *LYZ*, *LILRB*, or *ITGB2* are expressed **(F)** Dotplot indicating the distribution of expression and fraction of cells in various cell type for genes up-regulated (red) or down-regulated (blue) in responders vs non-responders to combination therapy as indicated in **(D)**.

Single-cell RNA sequencing analyses allow a more comprehensive characterization of the tumor environment and evaluation of the cell interplay. Analyses of more than 300 thousand cells of adnexal ovarian tumor tissue from 29 patients of the MSK cohort (29) allowed a clear separation between major cell type populations by clustering and nonlinear projection (UMAP) (**Figure 5A**, **Supplementary Figure S7**). In contrast to cell types from the tumor microenvironment, tumor cells showed a clear separation between BRCAness and noBRCness samples (**Figure 5A**). To further investigate the effect of BRCAness on the tumor microenvironment we compared the distribution of major cell types between BRCAness and noBRCAness samples. We did not observe any significant differences for the major cell types between these patient groups (**Supplementary Figure S8**) but in a summarized analysis (**Figure 5A**) the proportion of myeloid cell was slightly higher and the proportion T/NK cells was slightly lower in BRCAness compared to noBRCAness. Furthermore, we investigated the differential expression of immune response marker genes (**Supplementary Table S7**) in the BRCAness versus the noBRCAness group by pseudobulk analysis with DESeq2. Interestingly, 21 genes have been found significantly upregulated in BRCAness compared to noBRCAness including immune checkpoints such as *CD274* and a number of antigen processing and presentation genes (**Supplementary Figure S9**). Because cells from the suppressive environment have a major impact, we focused on the myeloid cell compartment and demonstrated that the majority of these cells were macrophages, and we identified subpopulations based on most dominant marker genes, including CD169 (SIGLEC1) macrophages, CX3CR1 macrophages, and MARCO macrophages (**Figure 5B**). One described hallmark marker of TAMs is TREM2, which has been identified as an attractive target for cell depletion therapy and is being tested in an ongoing clinical trial (64). Notably, the expression patterns of *TREM2* and BRCAness are very similar, showing high expression in all macrophage subtypes and, to a lesser extent, in monocytes (**Figure 5B**). To search for further genes with similar expression patterns in myeloid subpopulations, we analyzed known tumor-associated macrophage and monocyte marker genes (65). As indicated by this analysis, *C1QA* showed a similar but even more pronounced expression pattern than *TREM2* (**Figures 5B, C**). C1QA was also recently described as a surrogate marker for the CD68+CD163+ macrophage subset (66). We found that several genes are highly expressed in macrophages (**Supplementary Figure S10**) and that, based on marker genes, a polarization towards M2 macrophages occurs (**Supplementary Figure S11**). The immune suppressive effect of tumor associated macrophages were underscored by a number of myeloid immune checkpoint genes, which show a worse effect on overall survival (hazard ratio>1) in the TCGA cohort (**Supplementary Figure S12**).

Since our main goal was to predict vulnerability to combination immunotherapy we took advantage of the availability of gene expression data from a clinical trial (TOPACIO) and could identify 7 upregulated genes and 22 down regulated genes between responder and non-responder to PARPi-immune checkpoint inhibition combination therapy (niraparib and pembrolizumab) (**Figure 5D**). We next analyzed in which cell types these genes are generally expressed using the results from our single cell RNA-sequencing data analyses from the MSK cohort and found a number of downregulated genes in responders, such as *LYZ*, *LILRB4*, and *ITGB2*, were most highly expressed in myeloid cells (macrophages), *LILRB4* in dendritic cells, and integrin subunit beta 2 (*ITGB2*) in other cell types, such as T/NK cells (**Figures 5E, F**). *ITGB2* was also found correlated in the TCGA cohort with estimated M2 macrophages and CD8 T-cell infiltration (**Supplementary Figure S13**).

Interestingly, we identified various ligand−receptor interactions with expressed ligands in tumor cells and respective receptors expressed in tumor-associated macrophage subsets using CellPhoneDB (59) (**Supplementary Figure S14**). The growth arrest-specific protein 6 (GAS6) – AXL tyrosine kinase (AXL) interaction, for example, which are both associated with poor outcome, have already been evaluated in clinical trials in ovarian cancer by inhibiting their interaction (67). LILRB1 and LILRB2 expressed in macrophage subsets were found to interact with the nonclassical human leukocyte antigen HLA-F expressed in cancer cells. Blocking macrophage colony-stimulating factor CSF1 and its receptor CSF1R axis and several drugs that target these factors have been under investigation (68).

These observations summarized together suggest that TAMs may not only play a role in immunotherapy alone but are also essential in informing about therapy response when combined with PARP inhibitors.

### 3.6 Analyses of an independent cohort indicate vulnerability to combination immunotherapy

**Figure 6:**
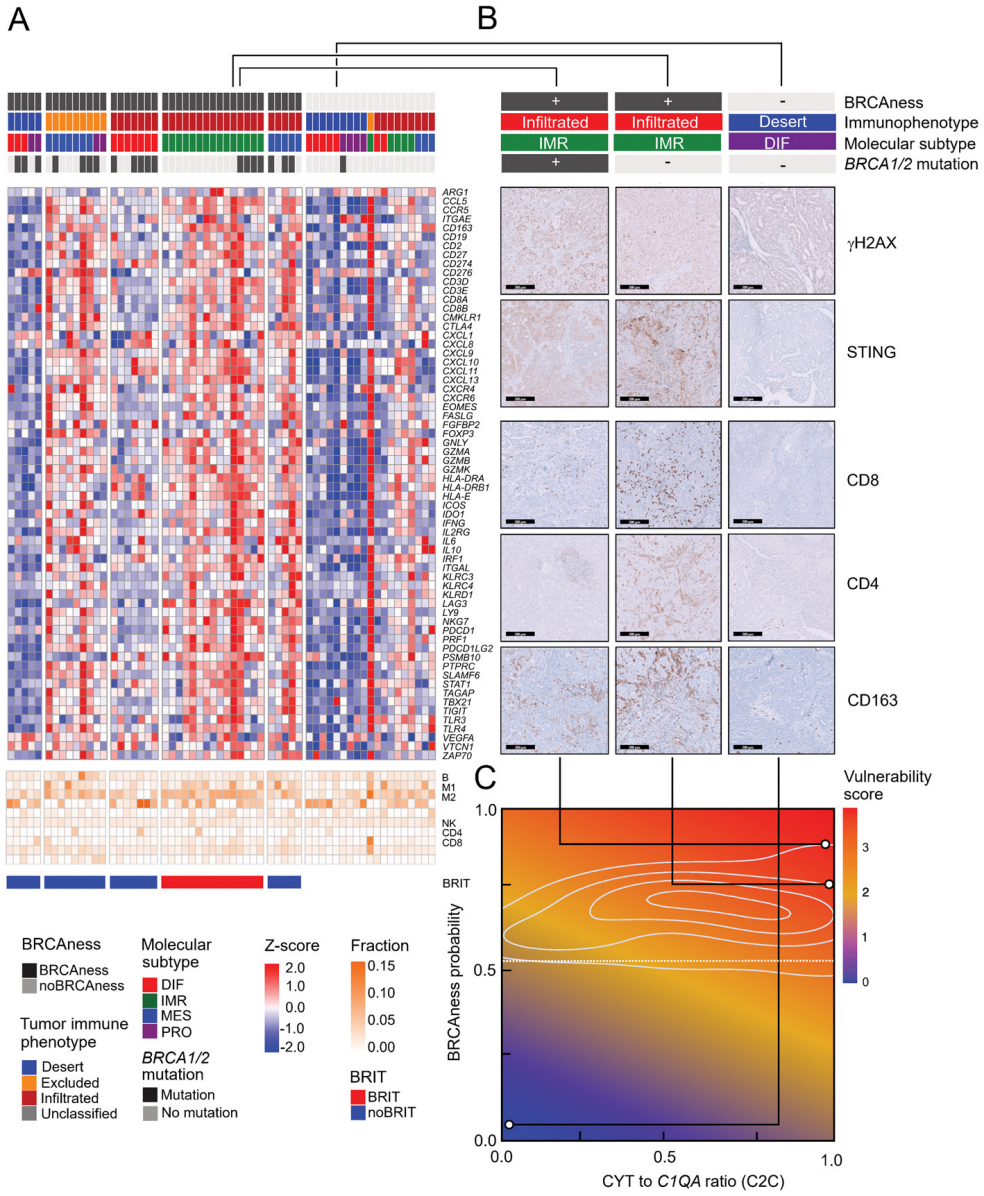
Expression profiles in the MUI cohort, immunohistochemistry validation, and vulnerability map **(A)** Heatmap of z-scores log2(TPM+1) expression of immune related genes and fraction of tumor infiltrating immune cells assessed with quanTIseq in all samples (n=60) from the MUI cohort categorized by BRCAness, tumor-immune phenotype, molecular subtype and BRCA1/2 mutation. **(B)** Immunohistochemistry images stained for CD8, CD4, CD163, γH2AX, and STING for three selected patients from the MUI cohort. Two BRIT samples one with a BRCA1 mutation and one without and one other sample without BRCAness, a deserted tumor-immune phenotype and a differentiated molecular subtype. **(C)** Vulnerability map showing the ratio between cytolytic activity CYT and C1QA (C2C) on the x-axis and the BRCAness score on the y-axis colored by the vulnerability score. The three selected samples were mapped to the vulnerability map based on their CYT to C1QA ratio (C2C) and BRCAness score.

To validate the results, we performed RNA sequencing analyses of an HGSOC cohort of patients from Medical University Innsbruck (n=60). Stratification of these patients resulted in very similar expression patterns evident from a number of immune marker genes, which were highly expressed in the BRCAness immune type patient group (BRIT) (**Figure 6A**). To further characterize immune infiltrates in different patient groups, we performed immunohistochemistry analyses on ten selected samples for various markers and highlight the results from three patient samples. One BRIT tumor sample showed high γH2AX activity, STING activation, CD8+ T-cell infiltration, CD4+ T-cell infiltration, and strong CD163+ tumor-associated macrophage populations (**Figure 6B**, left panel). These effects were even more pronounced in one sample with no detected *BRCA1* or *BRCA2* mutation, underscoring the importance and validity of predicted BRCAness (**Figure 6B**, middle panel). Another tumor sample with no BRCAness, a desert tumor-immune phenotype, and a differentiated molecular subtype was used as a negative control, and in fact, no activity for any of the tested markers was observed (**Figure 6B**, right panel). To better address the potential for combination immunotherapy response, we again took advantage of data from the TOPACIO trial and, based on the clinical response, trained a logistic regression model and learned weights for three surrogate variables: MutSig3 as an indicator for BRCAness, average expression of *PRF1* and *GZMB* as indicators for cytolytic activity, and expression of *C1QA* as an indicator for tumor-associated suppressive macrophages. Based on the HGSOC samples from TCGA, we developed a two-dimensional vulnerability map, with the ratio of cytolytic activity and *C1QA* expression as one variable (C2C) and the BRCAness prediction probability as the other variable. The vulnerability score is indicated by color (**Figure 6C**). When applied to the selected examples from the MUI validation cohort, these differed significantly for areas with high vulnerability scores (indicating response to combination immunotherapy) compared to the negative control with low vulnerability scores (**Figure 6C**). Furthermore, we observed a significant difference in overall survival between patients with high and low vulnerability scores (p<0.001, HR = 0.47, 95% CI 0.33-0.66) in the TCGA HGSOC cohort (**Supplementary Figure S15**), indicating a positive association of a high vulnerability score with longer overall survival. For patients in the MUI validation cohort, no significant difference in overall survival (p=0.368, HR = 0.78, 95% CI 0.45-1.34) could be revealed (**Supplementary Figure S16**). To enable the characterization of newly diagnosed HGSOC samples based on RNA sequencing data, we developed an easy-to-use R package (OvRSeq), which allows us to not only estimate the parameters to determine the vulnerability score (and generate the vulnerability maps) but also comprehensively annotate the sample for BRCAness, tumor-immune phenotype, molecular subtype, estimate immune infiltrates, enrichment of immune-related signatures, and individual marker genes. This also includes other clinically relevant parameters, such as the angiogenesis score we previously defined, which might be useful for the prediction of anti-VEGF therapy (69). The web application (https://ovrseq.icbi.at) allows the generation of summary information as a report of individual samples (**Supplementary Figure S17**).

The developed application should ultimately be useful to identify vulnerabilities and support clinical therapy decisions for HGSOC patients.

## 4 Discussion

Here, we described how genomic instability in HGSOC affects the tumor immune environment and the consequences and vulnerabilities of combination immunotherapy combining PARP inhibitors with immune checkpoint inhibitors. A particular status in which patients respond well to PARP inhibitors and platinum-based chemotherapy is given when genes of the HRR pathway such as *BRCA1* or *BRCA2* are mutated. Genomic scars are consequences of HRD and are used to define an HRD score, often measured by established commercial assays, which allows the assignment of a responsive status beyond *BRCA1* and *BRCA2* mutations. The applicability and associated cutoff values for different assays and cancer types are under discussion, as the HRD algorithm has been used in clinical studies including different cancer types, such as breast cancer and ovarian cancer (46, 70, 71). Genomic scars are predictive but do not allow direct functional interpretation, whereas gene expression signatures could be an alternative in this regard. Very few approaches have associated gene expression with HRD status (18, 19, 62). Whereas a sixty-gene signature (18) and a two-gene signature (*CXCL1*, *LY9*) (19) have focused on microarray data, a recent approach using RNA sequencing data identified a 249-gene signature to predict HRD (62). We observed a number of overlaps with our 24-gene BRCA signature and a high concordance of signature scores in our training (TCGA) and validation (MUI) cohorts, indicating the reliability of our approach. This was underscored by comparison with mutational signature 3 (SigMA) in an independent cohort. The performance of the BRCAness classifier is reasonable, with AUC=0.91 (10-fold cross-validation) and positive predictive value for validation on both bulk RNA sequencing in the validation cohort (MUI) (PPV=0.86) and sample-wise single-cell RNA sequencing data (PPV=0.87).

There is evidence that BRCA1/2-mutated tumors exhibit significantly increased CD8+ TILs (22), although in breast cancer, differential modulation between *BRCA1* and *BRCA2* mutations in the tumor immune microenvironment has been found (72). We found a significant association between BRCAness and several immune-regulated signatures and evidence that several signaling pathways and processes known to modulate the immune system are activated by *BRCA1* mutations or a BRCAness-related phenotype, such as JAK-STAT signaling or an interferon type I response, which are activated by free double-stranded DNA in the cytoplasm of tumor cells via the cGAS-STING pathway and affect dendritic cells (23–25). By expression and immunofluorescence analyses of ovarian cancer cell lines and by treatment with PARPi, we demonstrated that this axis is actually activated. Notably, the STAT3 pathway, which is activated by PARP inhibition, may, however, mediate treatment resistance by promoting the polarization of protumor TAMs, which could be overcome by STING agonism (26). STING, CSF1R, SREBP-1, and VEGFA might also be targets to overcome resistance to PARPi-immunotherapy combinations (73). The upregulation of many chemokines and chemokine receptors indicates that BRCAness tumors are actively involved in immune cell attraction and interaction. For example, CCL5 produced by tumor cells or *CXCL9* and *CXCL10* also expressed by tumor-resident myeloid cells determine effector T-cell recruitment to the tumor microenvironment (74). We detected significant upregulation of *CCL5* and *CXCL10* by PARP inhibition, which was also identified as a downstream target of STING (24). Another interesting chemokine that is strongly upregulated in cancer cell lines, particularly by olaparib treatment, is *CCL20*. CCL20 could be associated with cancer metastasis and progression by interacting with its cognate receptor CCR6 in an ovarian cancer mouse model. However, the higher expression in the myeloid cell compartment, as evident from single-cell analyses, overlies the intrinsic tumor effect.

One of our basic hypotheses was that samples with BRCAness respond better to PARPi therapy and that hot tumors with an activated immune milieu respond better to immune checkpoint inhibition, as has been shown, for example, in melanoma for the activated IFNG pathway (75). However, when we compared the BRCAness immune type (BRIT) with other samples, we observed by using deconvolution approaches that suppressive cell types such as M2 macrophages, MDSCs, and Tregs were more abundant. In particular, TAMs could be a major factor together with low mutational burden, abnormal neovascularization, altered metabolism, and failure to reverse T-cell exhaustion for the limited immunotherapy response in ovarian cancer (76). By using single-cell RNA sequencing data analyses of adnexal cancer tissue, we demonstrated that myeloid cells are the most abundant immune cells, and the majority were characterized as TAMs and rather polarized towards M2-like macrophages compared to classical (M1-like) macrophages, although these better described as continuum of different stages than isolated cell types. A majority of these TAMs are immune suppressive, as indicated by *TREM2* expression. TREM2 is a promising therapeutic target for TAM depletion (68). Inhibition of TREM2 has been shown to improve the anti-PD1 response in various mouse models and is currently being investigated in a clinical trial (64, 77). Another recent study underscored the role of TAMs and demonstrated that specifically, the Siglec-9-positive TAM subset is associated with an immune-suppressive phenotype and adverse prognosis in HGSOC patients (78).

Interestingly, a previous work using cyclic immunofluorescence highlighted the role of exhausted T cells in the response to niraparib/pembrolizumab. In responders, particularly in extreme responders, frequent proximity between exhausted T cells and PD-L1+ (CD163+, IBA1+, CD11b+) TAMs was observed (9). Noticeably, based on the selected marker expression, we observed an overlap with the CD169/SIGLEC-1 macrophage cluster (**Supplementary Figure S10**). In addition, in patients who responded to this combination therapy, we identified a number of downregulated genes that were also highly expressed in TAMs, such as *LYZ*, *LILRB4*, and *ITGB2*. Whereas lysozyme (LYZ) is an antimicrobial ligand and is involved in central macrophage function and is therefore nonspecifically and highly expressed, LILRB4 is an immune checkpoint on myeloid cells, indicating a more regulatory role. High expression of the integrin *ITGB2* was previously shown to be associated with poor survival outcome (79), underscoring that high expression in TAMs is crucial. In contrast, *ITGB2* is also associated with CD8+ T cells, as it encodes the beta chain of the LFA-1 protein, which has been shown to be essential in the assembly of the immune synapse or to influence lymphocyte extravasation and T-cell recruitment to the tumor and is regulated by GDF-15 (80).

Because stratification of patients based on gene expression in our validation cohort was very similar to the analysis on the TCGA cohort, we set out to adapt our hypothesis and also include elements of the suppressive environment. Already, it was shown that regulatory T cells (Tregs) are an important component of the suppressive milieu and are associated with unfavorable survival outcomes in ovarian cancer (81, 82). We performed immunohistochemistry analyses using FOXP3 and CD163 antibodies in the validation cohort and found very pronounced macrophage infiltration (CD163) but hardly Treg infiltration (FOXP3) into the tumor site in some samples. The results of the single-cell RNA sequencing analyses and the fact that various TAM marker genes were associated with poorer overall survival also suggest that TAMs play a more dominant role in ovarian cancer.

While infiltration of various cell types from the adaptive immune system (83) and other markers, such as tumor mutational burden (TMB) (84) or IFNG signature (75), have been associated with good prognosis and immunotherapy response in various cancer types, the suppressive immune environment with tumor-supportive CD68+CD163+ macrophages is becoming more important (66). Accordingly, a signature of the immune activation ratio of CD8A/C1QA has been found to be prognostic and predictive for immunotherapy response (66). We considered the mean *PRF1* and *GZMB* expression as a proxy for cytolytic activity (45) as predominantly exerted by cytotoxic T lymphocytes. The specific expression pattern of *C1QA* on TAMs was comparable to that of *TREM2* but at a much higher level. Therefore, we also used the member of the complement system *C1QA* as a surrogate for TAMs and the suppressive tumor immune environment and finally built a ratio of cytolytic activity (CYT) to the expression of *C1QA* (C2C), indicating the pro- and antitumoral balance of the immune environment. Finally, to build a predictive algorithm for combination therapy response, we included both C2C on the one hand and BRCAness on the other hand into one model. Since HRD measured with companion diagnostic tests is not able to predict all PARPi responders, as shown in several clinical trials, and since PARPi treatment can activate a number of immune-related pathways even in situations with proficient HRR, which is also underlined by our *in vitro* analyses, this model is considered to be relevant for combination immunotherapy.

Our studies have some limitations in that the training and validation patient cohorts were retrospective studies, and RNA sequencing was performed at a later time point. Additionally, only a limited number of patients who received combination therapy could be included; therefore, the conclusion about the predictive power for the treatment is limited and requires further validation in larger cohorts. One component that was not considered in this study is malignant ascites, which has been shown to contain various cell types, such as macrophages, many soluble factors and cytokines, that influence the protumorigenic phenotype and promote metastatic spread of HGSOC through transcoelomic dissemination (85).

In conclusion, our approach using RNA sequencing data to comprehensively characterize both genome instability and the tumor immune environment enabled us to stratify HGSOC patients. Further analyses indicate that suppressive TAMs in the tumor immune microenvironment may play an essential role in understanding why receiving (combination) immunotherapy shows limited efficacy in ovarian cancer. Based on multiple datasets, we have developed a methodology and corresponding easy-to-use diagnostic application (https://ovrseq.icbi.at) and an R package OvRSeq that uses RNA sequencing data not only to comprehensively characterize newly diagnosed HGSOC patients but also to inform therapy response. Ultimately, this approach will be very useful to obtain comprehensive information about the phenotype of a tumor sample, support clinical decisions, and stimulate further research.

## Data Availability

The original contributions presented in the study are included in the article/supplementary material. RNA sequencing data can be found at Gene Expression Omnibus (GEO) (https://www.ncbi.nlm.nih.gov/geo/query/acc.cgi?acc=GSE237361). The raw RNA sequencing data from the MUI cohort cannot be made publicly available due to data protection restrictions and are available on request from the corresponding author.
