# Supplementary material for: Integrated immunogenomic analyses of high-grade serous ovarian cancer reveal vulnerability to combination immunotherapy
Source: Front Immunol. 2024 Nov 28;15:1489235. doi: 10.3389/fimmu.2024.1489235 (PMC11634877; doi:10.3389/fimmu.2024.1489235)
Supplement: Supplementary file 1 [file DataSheet1.docx]

Supplementary Material

# Supplementary Figures and Tables

## Supplementary Figures


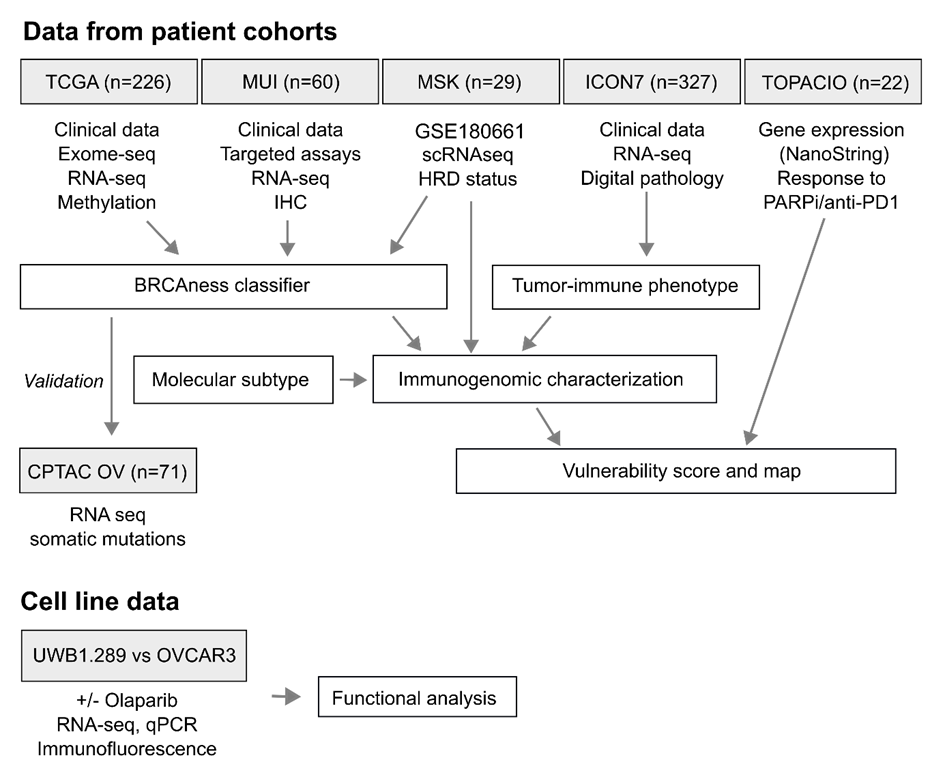


**Figure S1.** Workflow of analyses and ovarian cancer patient cohorts with corresponding data sets used for immunogenomic characterization of patient samples and ovarian cancer cell line data. IHC, immunohistochemistry; OV, ovarian cancer; HRD, homologous recombination repair deficiency; PARPi, PARP inhibitor; qPCR, quantitative reverse transcription polymerase chain reaction; scRNAseq, single cell RNA sequencing; CPATC, Clinical Proteomic Tumor Analysis Consortium; TCGA, The Cancer Genome Atlas; MUI, Medical University of Innsbruck; GSE, Gene Expression Omnibus Identifier, MSK, Memorial Sloan Kettering Cancer Center.


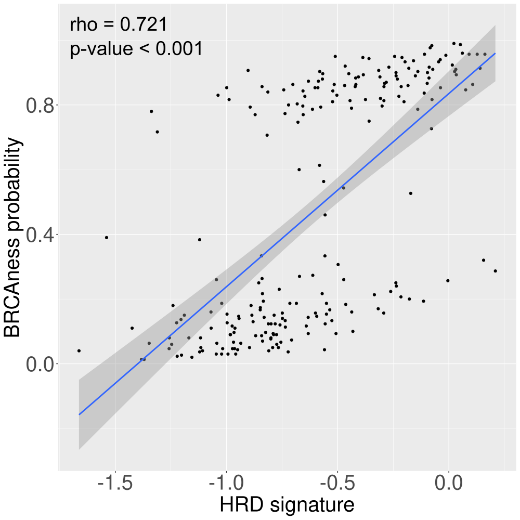

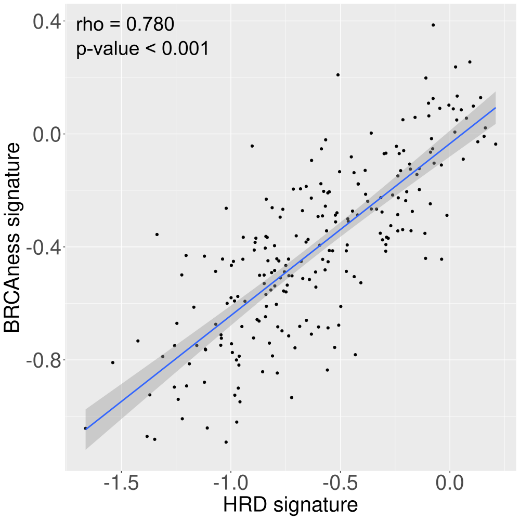


**Figure S2.** Correlation of different BRCAness and HRD-parameter in the TCGA cohort (n=226). BRCAness prediction probability and HRD signature (1) (left), BRCAness signature and HRD signature (1) (right).


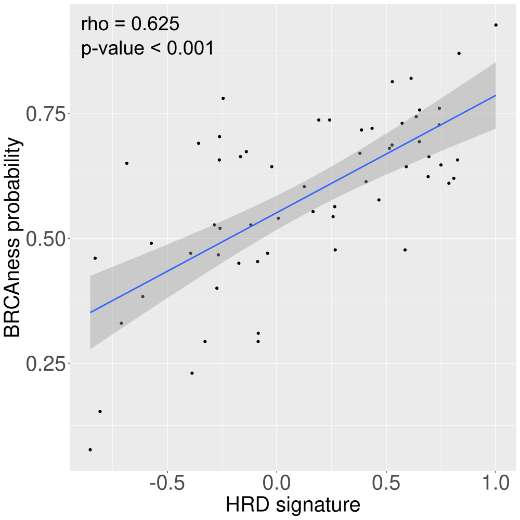

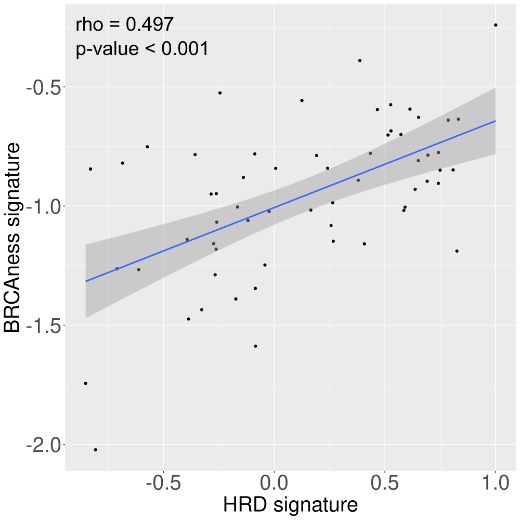


**Figure S3:** Correlation of different BRCAness and HRD-parameter in the MUI cohort (n=60). BRCAness prediction probability and HRD signature (1) (left), BRCAness signature and HRD signature (1) (right).


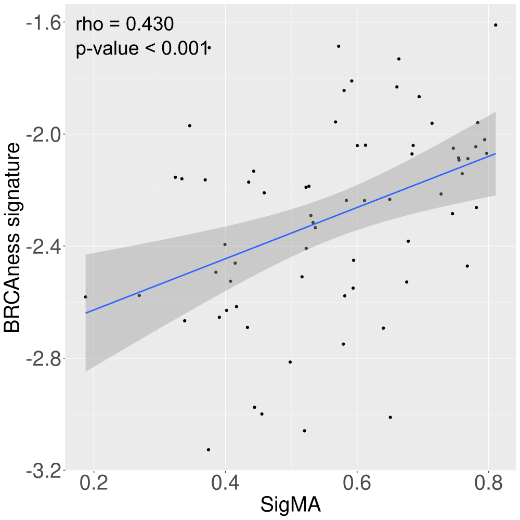


**Figure S4:** Correlation of the BRCAness signature and SigMA (Mutational signature 3) in the CPTAC-OV cohort (n=71).


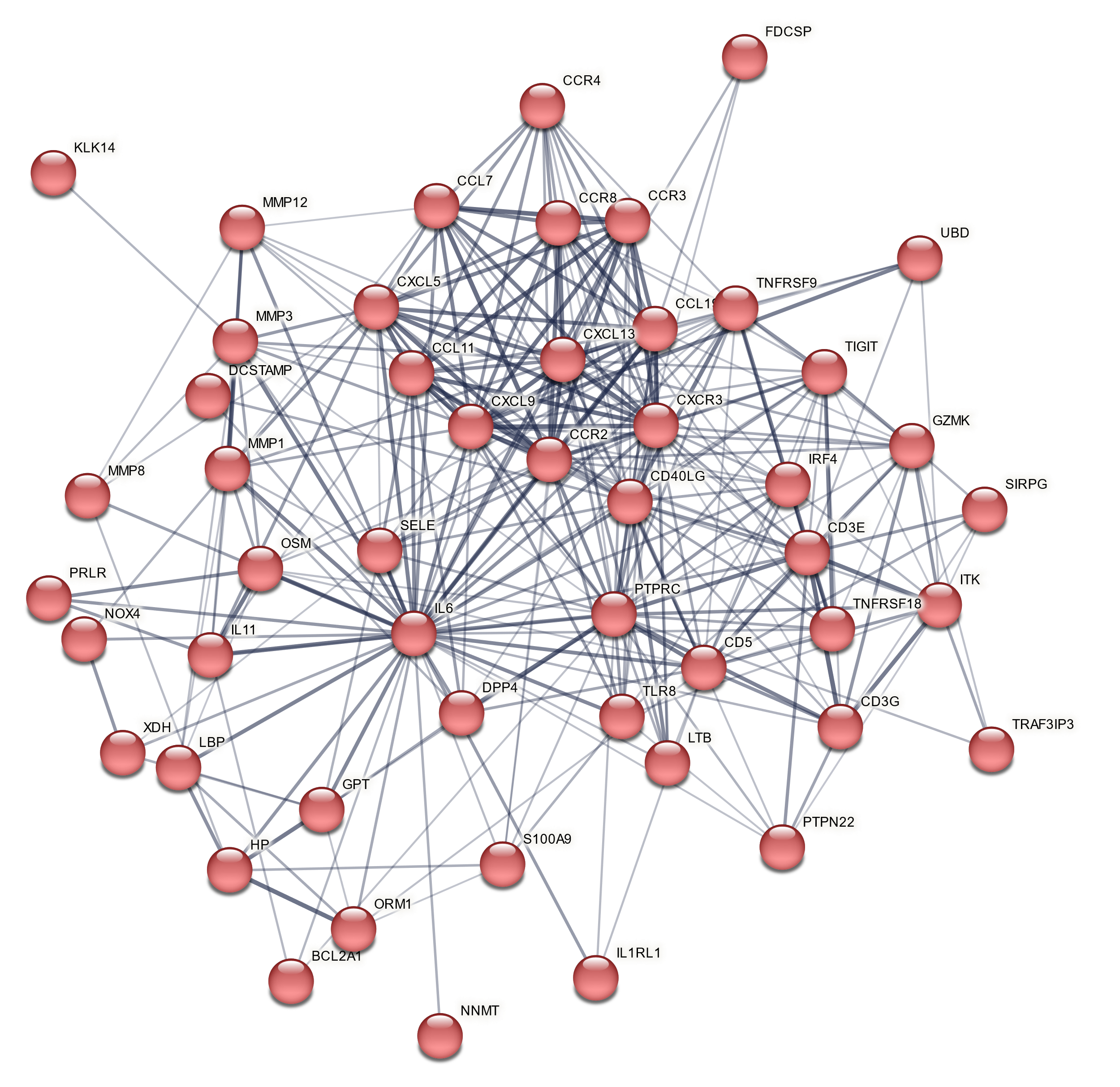


**Figure S5:** Network cluster of genes associated with immune signaling pathways, which are upregulated in the BRCAness samples of the MUI cohort (n=60). MUI, Medical University of Innsbruck.

##
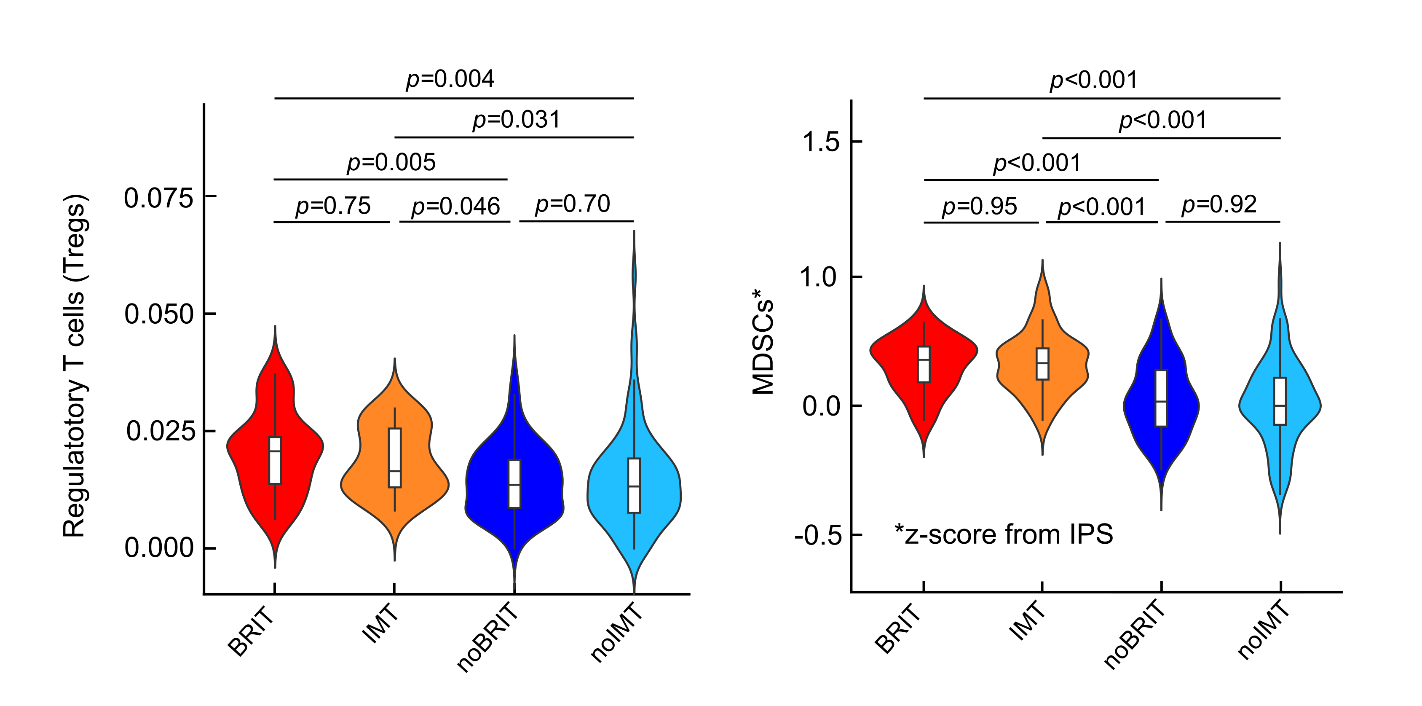


**Figure S6:** Distribution of estimated regulatory T cells (from quanTIseq analyses) and myeolid derived suppressor cells (MDSCs) (from immunophenscore analyses, IPS) for the different tumor subtypes BRCAness-immune type (BRIT= BRCAness & IMR & INF), immune type (IMT= noBRCAness & IMR & INF), noBRIT (BRCAness & not BRIT), and noIMT (noBRCAness & not IMT) with IMR is immunoreactive molecular subtype and INF is infiltrated tumor-immune phenotype. Following rejected null hypothesis by Krukal-Wallis test, pair-wise two-sided Dunn’s posthoc test with Benjamini-Hochberg adjusted p-values based on the false discovery rate (FDR) are indicated.

Tumor cells (Epithelial cells) Myeolid cells


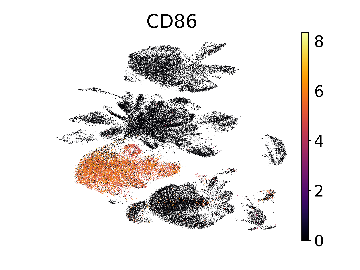

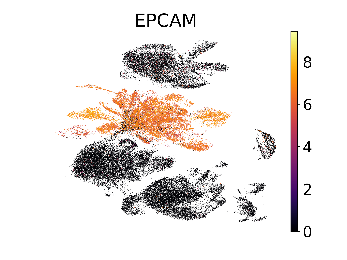


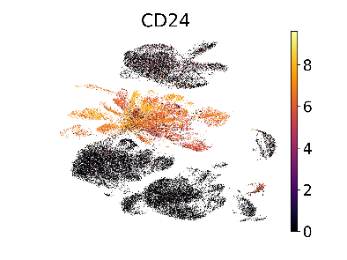

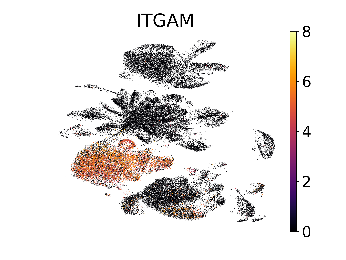


Fibroblasts B cells


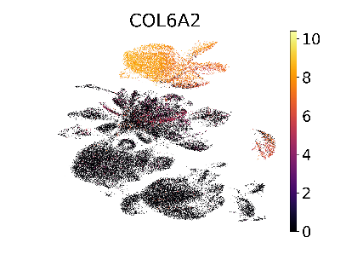

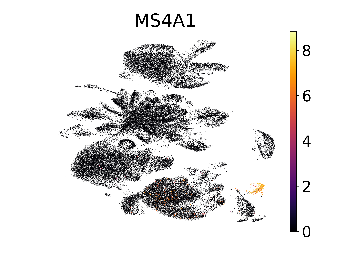

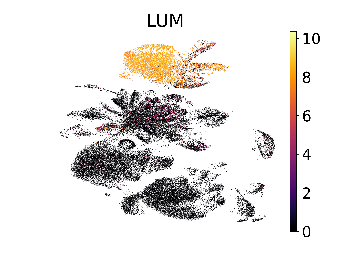

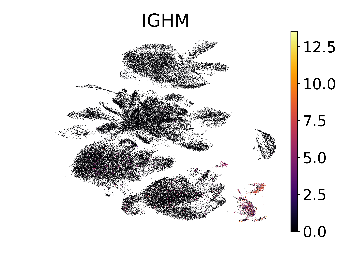


Plasma cells Dendritic cells (pDC)


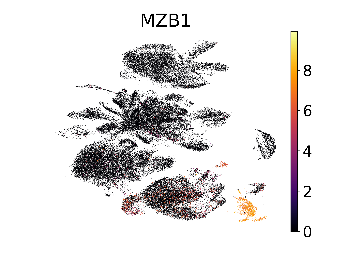

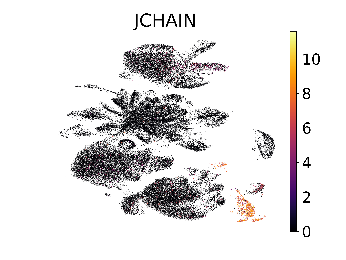


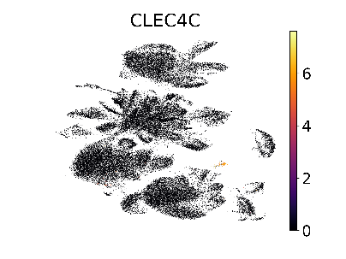

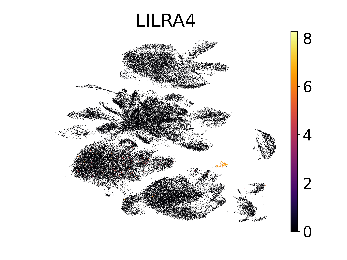


Mast cells Endothelial cells


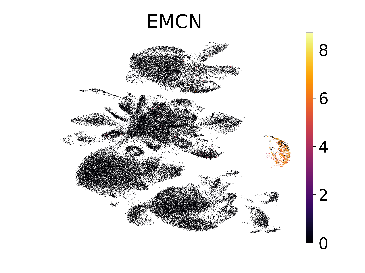

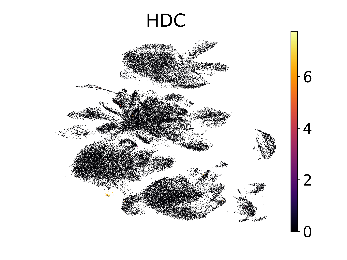


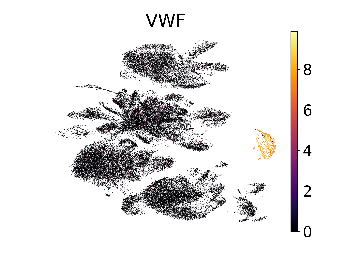

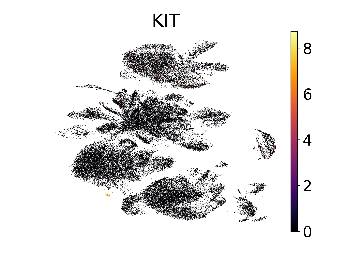


T cells/NK cells


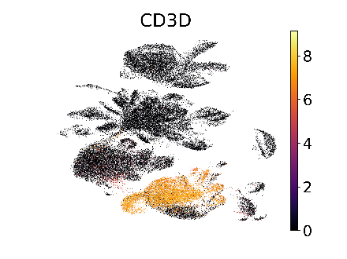


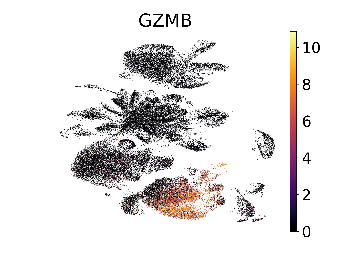

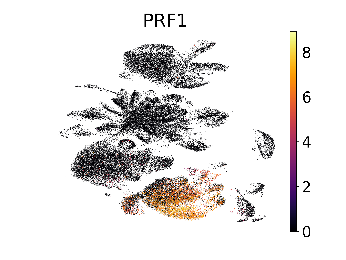


**Figure S7:** UMAP plots of selected cell type specific marker gene expression of major cell types of single-cell RNA sequencing analysis (MSK cohort n=29, GSE186601)


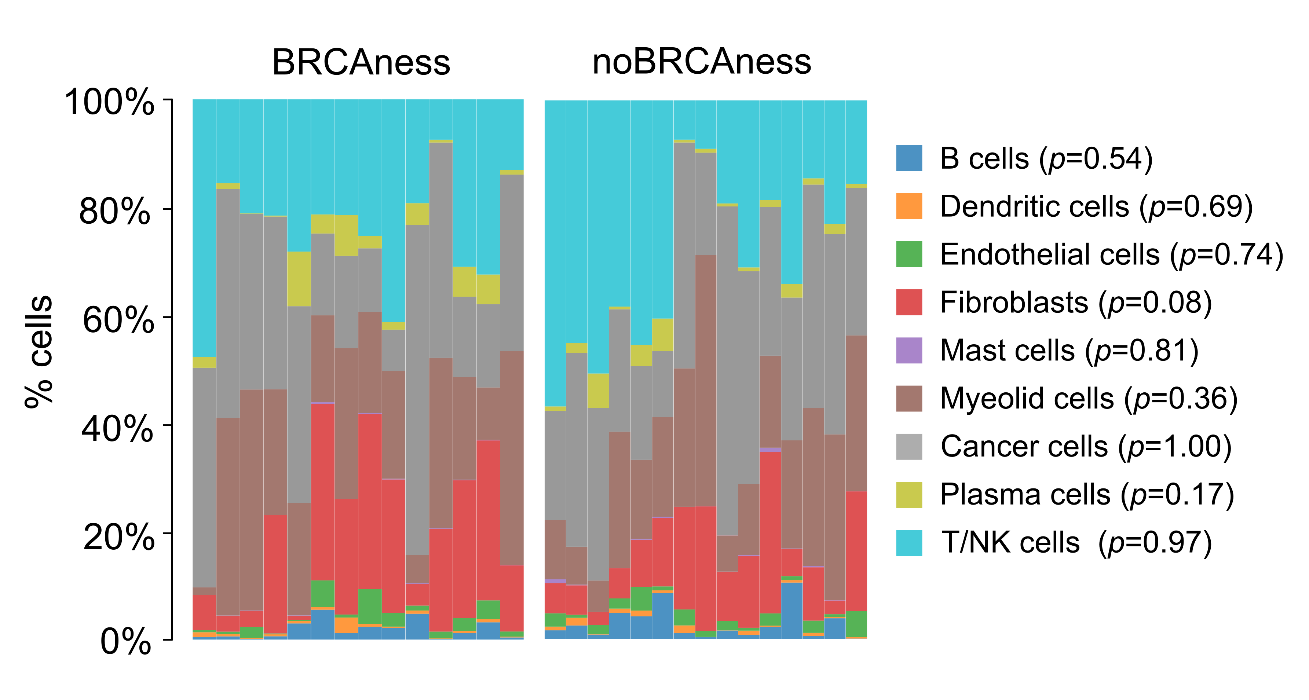


**Figure S8:** Distribution of major cell types in individual HGSOC adnexal samples of the MSK cohort from single-cell RNA sequencing analyses (n=29, GSE186601) and comparison between BRCAness (n=14) and noBRCAness (n=15) samples. Unadjusted p-values in brackets are from two-sided non-parametric Wilcoxon rank sum tests.


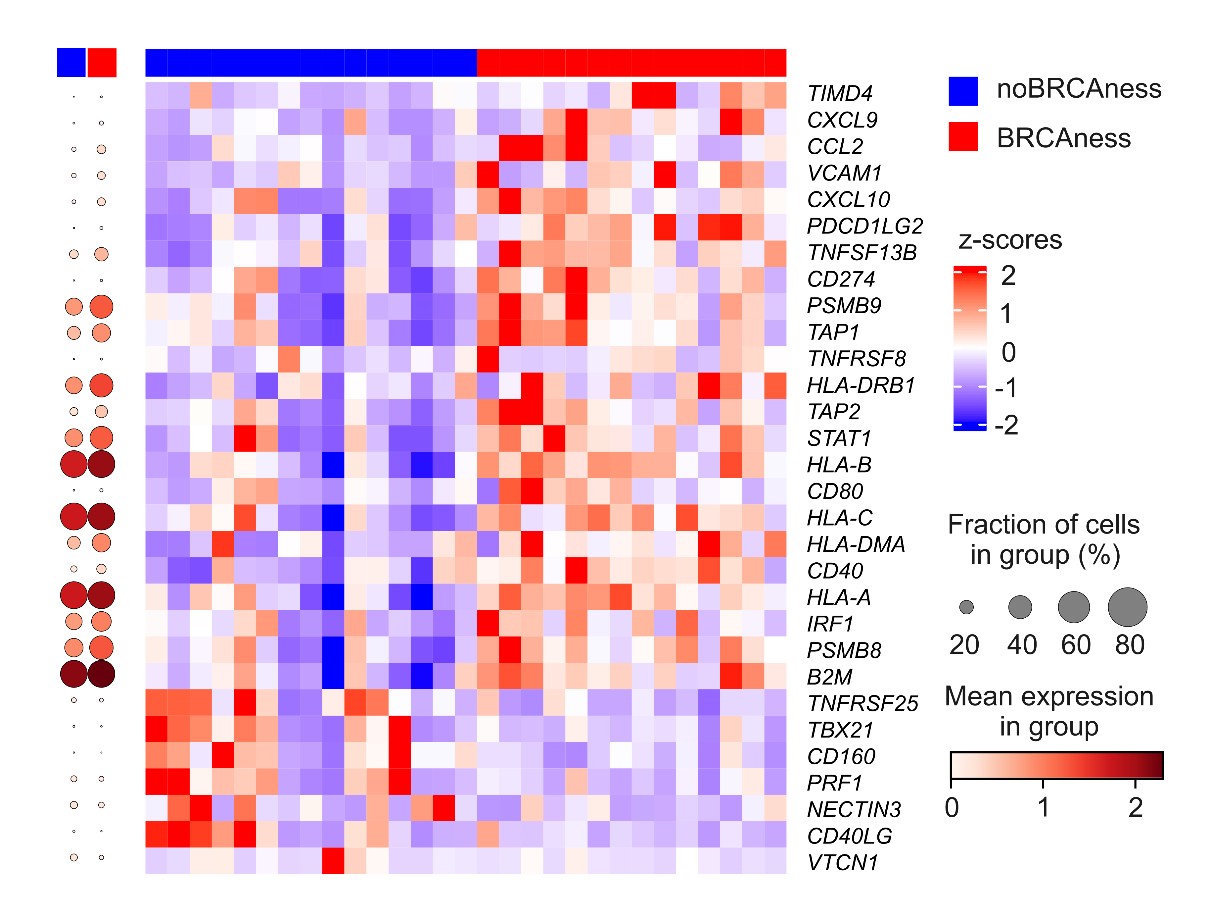


**Figure S9:** Significantly differentially expressed selected immune response marker genes (Table S7) between BRCAness (n=14) and noBRCAness (n=15) in adnexal samples of the HGSOC MSK cohort (n=29, GSE186601) by pseudobulk analyses of single-cell RNA sequencing data using DESeq2. Dotplot shows the mean expression and the proportion of cells expressing the gene in the BRCAness or noBRCAness group. Heatmap indicate expression (z-score) of genes in the respective sample. Only genes with p<0.05 and more than 1.5 fold change are shown.


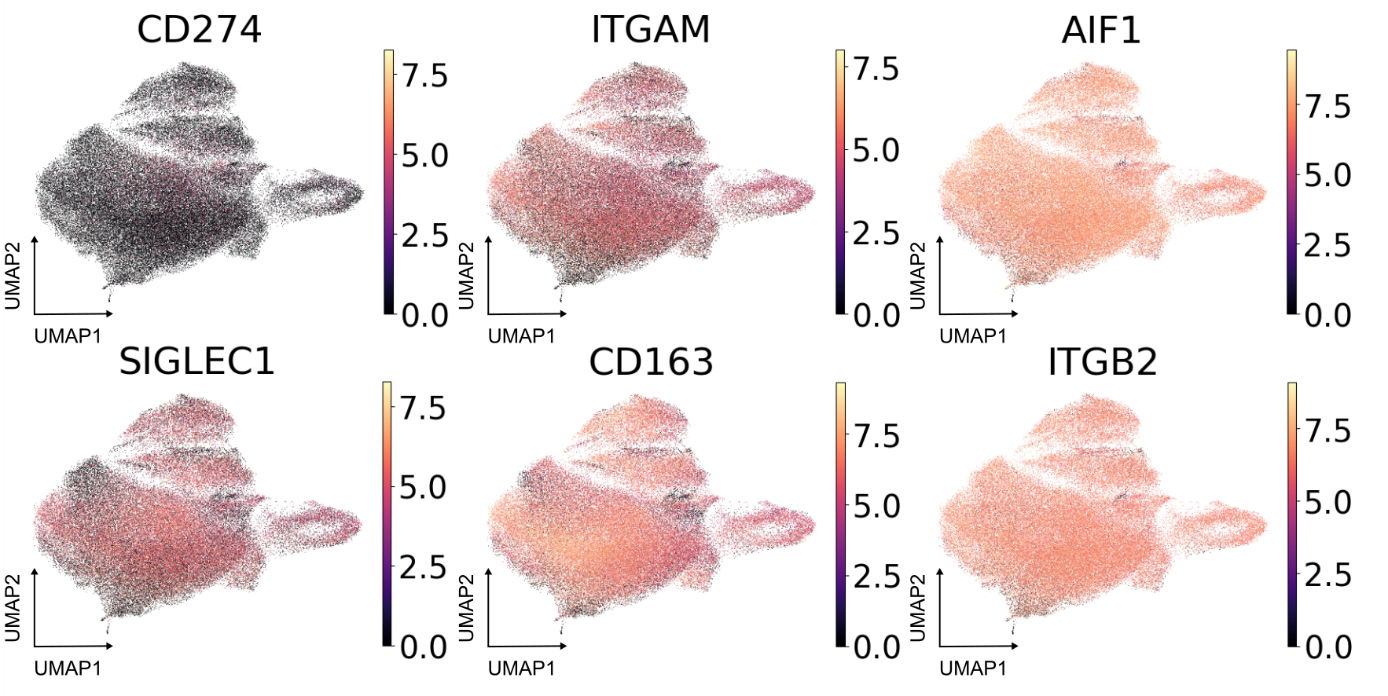


**Figure S10:** UMAP plots of selected macrophage associated genes in the myeloid cell compartment of single cell RNA-seq analyses.


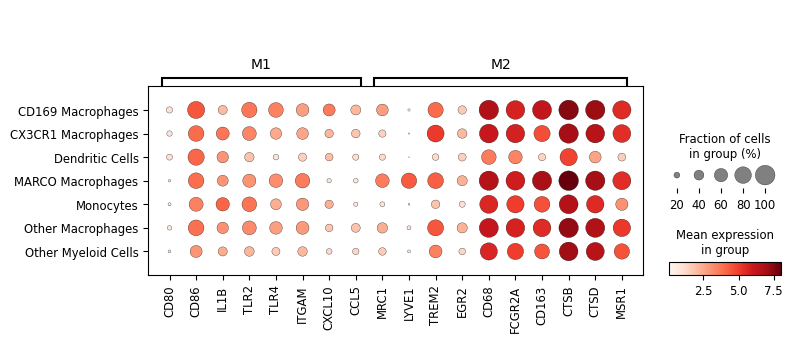


**Figure S11:** Expression of marker genes for macrophage polarization in the myeloid subgroups of the ovarian cancer microenvironment. Expression of M1 marker genes (left) and M2 marker genes right (M2) indicate a bias towards M2 macrophages, although discrimination is limited.


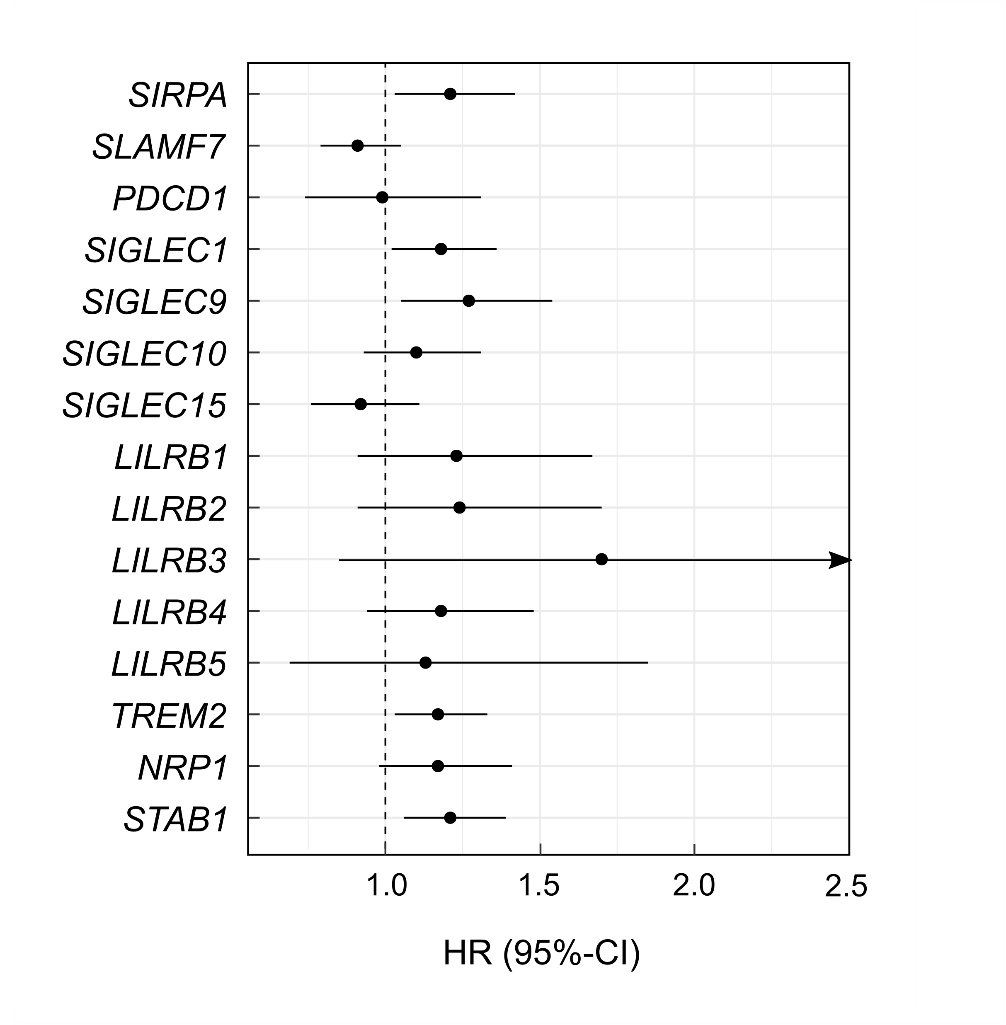


**Figure S12:** Forest plot showing the hazard ratio (HR) and the 95% CI of overall survival for selected genes associated with tumor associated macrophages in the TCGA cohort (n=226) using Cox regression and including BRCAness status as covariate. CI, confidence interval.


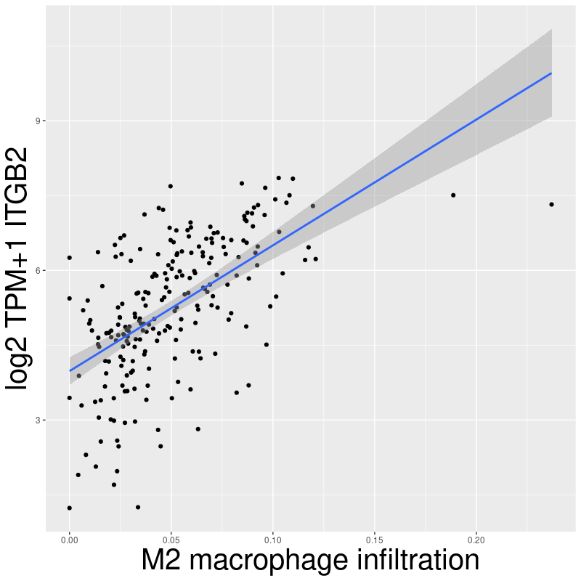

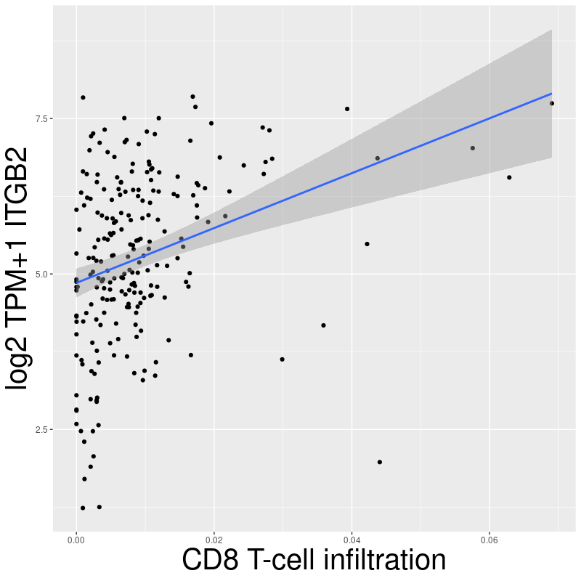


**Figure S13:** Correlation of M2 macrophage (left) and CD8 T-cell infiltration (right) and *ITGB2* expression in the TCGA cohort (n=226). TCGA, The Cancer Genome Atlas.


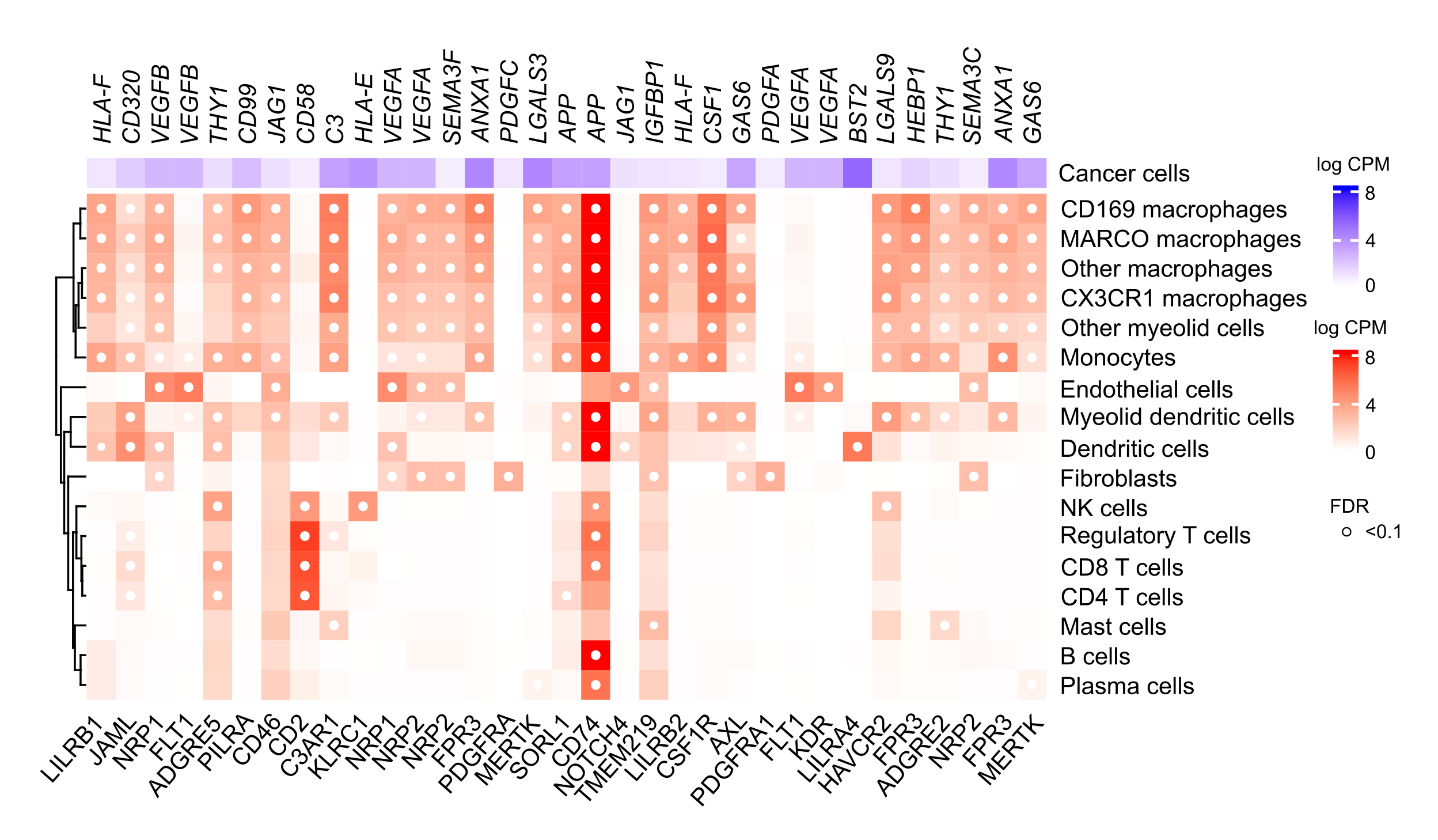


**Figure S14:** Receptor-Ligand interaction from CellPhoneDB analyses based on scRNAseq data from the MSK cohort (n=29). With expression of ligands on tumor cells (blue) and expression of respective receptors on immune cells (red). FDR<0.1 indicate significant interactions.


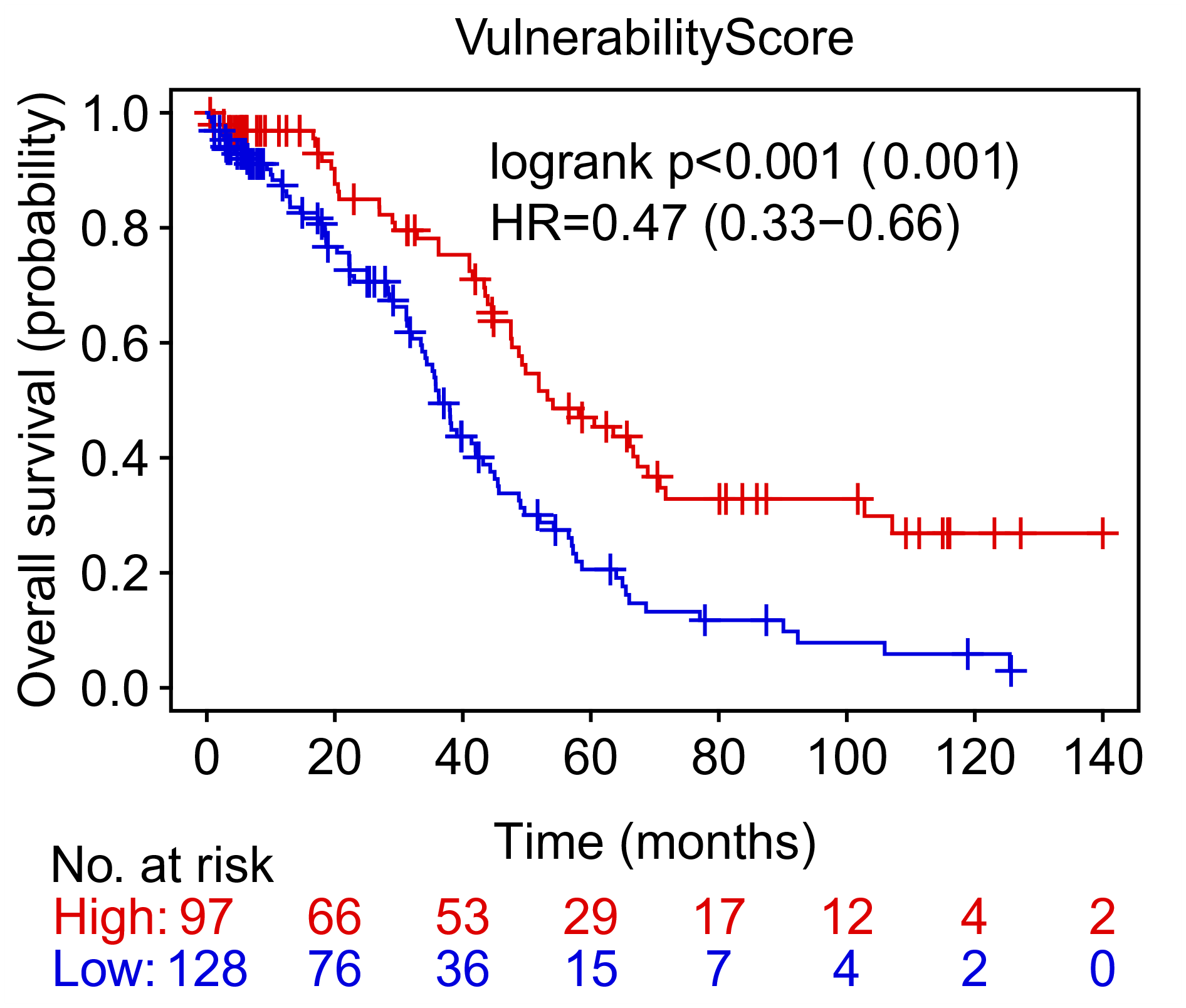


**Figure S15:** Kaplan Meier curve for the vulnerability score in the TCGA cohort. Patients were dichotomized using maximum logrank statistics/minimal p-value. Corrected p-values were calculated according to Altman et al. (2) and indicated in brackets. TCGA, The Cancer Genome Atlas.


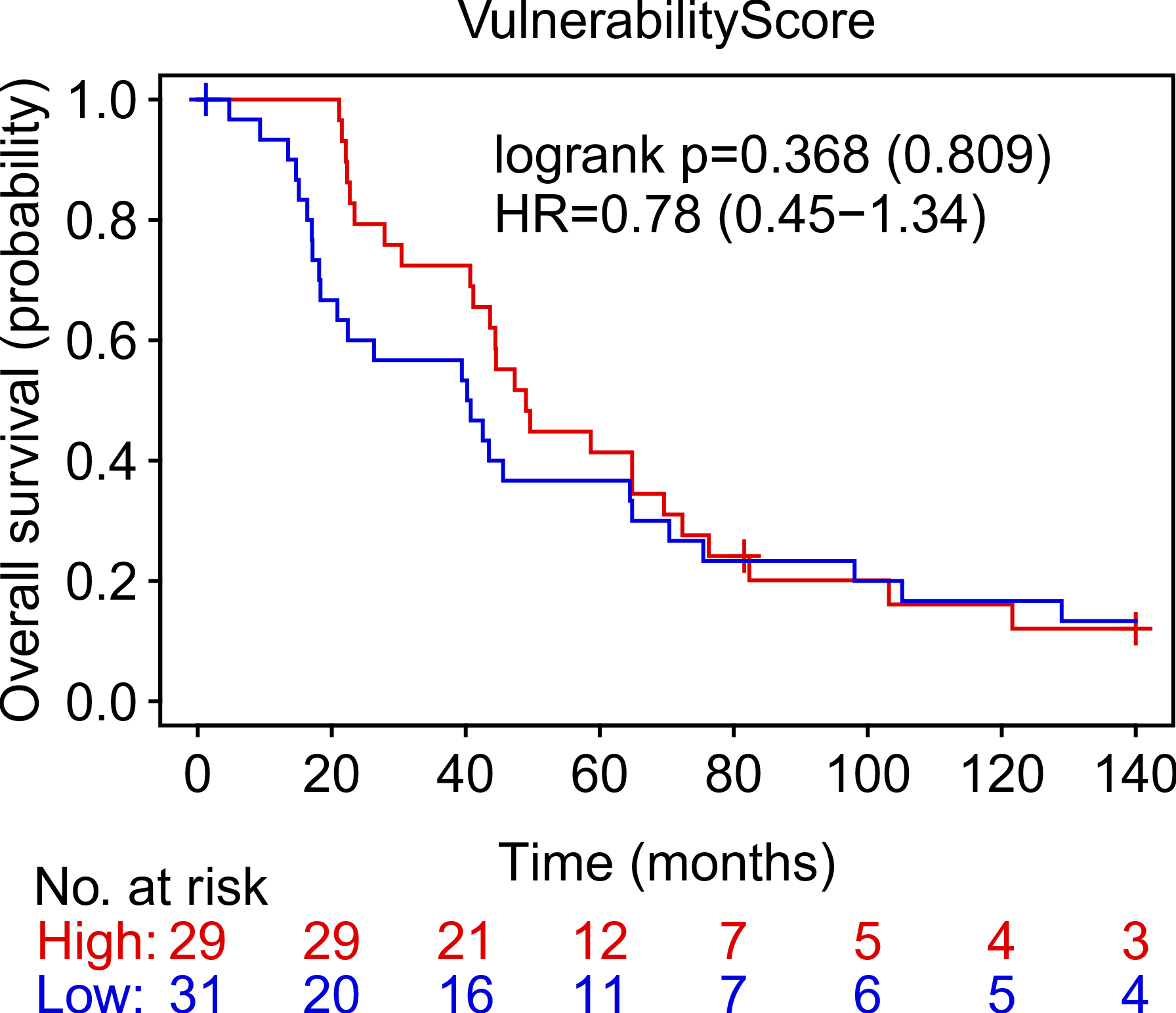


**Figure S16:** Kaplan Meier curve for the vulnerability score in the MUI cohort. Patients were dichotomized using maximum logrank statistics/minimal p. Corrected p-values were calculated according to Altman et al. (2) and indicated in brackets. MUI, Medical University of Innsbruck


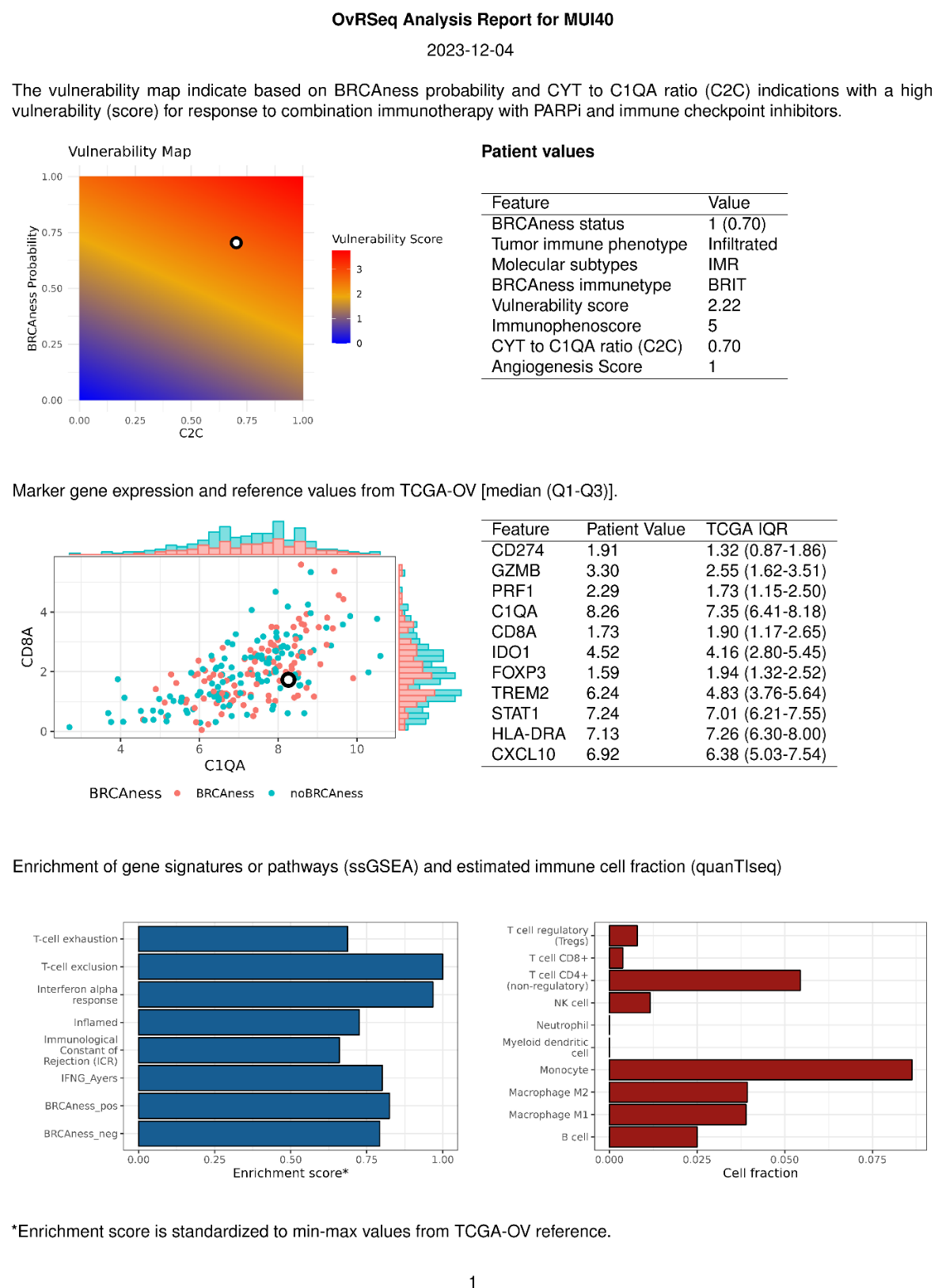


**Figure S17:** Report for comprehensive characterization including vulnerability map and score of one HGSOC patient sample from RNA sequencing data using the web application https://ovrseq.icbi.at or the R package OvRSeq (https://github.com/icbi-lab/OvRSeq).

## Supplementary Tables

**Table S1:** Patient characteristics of the high grade serous ovarian cancer (HGSOC) subset of the TCGA-OV cohort (n=226)

| Parameter | Range / Number of patients (%) |
| --- | --- |
| Age | 34-87 |
| FIGO Stage  1 and 2  3  4  na | 14 (6.2)  173 (76.5)  37 (16.4)  2 (0.9) |
| BRCAness  noBRCAness | 116 (51.3)  110 (48.7) |
| HRD-scores  >63  <=63 | 6-101  89 (39.4)  137 (60.6) |
| Patients with HRR mutation  BRCA1 mutations (germline/somatic)  BRCA2 mutations (germline/somatic)  other HRR mutations | 54 (23.9)  25/3 (11.1/1.3)  14/1 (6.2/0.4)  13 (5.8) |
| Tumor immune phenotype  Infiltrated  Excluded  Deserted  Unclassified | 79 (35.0)  27 (11.9)  97 (42.9)  26 (11.5) |
| Molecular subtype  Immunoreactive  Differentiated  Proliferative  Mesenchymal | 66 (29.2)  69 (30.5)  48 (21.2)  43 (19.0) |

**Table S2:** Patient characteristics of the HGSOC cohort from the Medical University of Innsbruck (MUI) (n=60).

| Parameter | Range / Number of patients (%) |
| --- | --- |
| Age | 32-83 |
| FIGO Stage  3  4 | 48 (80.0)  12 (20.0) |
| BRCAness  noBRCAness | 41 (68.3)  19 (31.7) |
| BRCA1 mutations  BRCA2 mutations | 16 (26.7)  4 (6.7) |
| Tumor immune phenotype  Infiltrated  Excluded  Deserted | 36 (60.0)  10 (16.7)  14 (23.3) |
| Molecular subtype  Immunoreactive  Differentiated  Proliferative  Mesenchymal | 20 (33.3)  17 (28.3)  8 (13.3)  15 (25.0) |

**Table S3:** Antibodies used for immunofluorescence staining of ovarian cancer cells from the UWB1.289 and OVCAR3 cell lines.

| Antibody | Catalogue  number | manufacturer |
| --- | --- | --- |
| ProLong® Gold Antifade Reagent with DAPI | #8961 | Cell Signaling Technology |
| STING (E9X7F) Rabbit mAb | #90947 | Cell Signaling Technology |
| Phospho-STING (Ser366) (E9A9K) Rabbit mAb | #50907 | Cell Signaling Technology |
| cGAS (E5V3W) Rabbit mAb | #79978 | Cell Signaling Technology |
| Phospho-Histone H2A.X (Ser139) (D7T2V) Mouse mAb | #80312 | Cell Signaling Technology |
| Anti-rabbit IgG (H+L), F(ab')2 Fragment (Alexa Fluor® 488 Conjugate) | #4412 | Cell Signaling Technology |
| Anti-mouse IgG (H+L), F(ab')2 Fragment (Alexa Fluor® 594 Conjugate) | #8890 | Cell Signaling Technology |
| ds DNA Marker Antibody (HYB331-01) | sc-58749 | Santa Cruz Biotechnology |

**Table S4:** Antibodies used for immunohistochemistry staining of patient derived FFPE tissue slides from the MUI cohort.

| Target | Antibody | Company |
| --- | --- | --- |
| γH2AX (DNA-damage) | AF2288 | R&D Systems |
| STING (cGAS_STING activation) | HPA038116 | Sigma Aldrich |
| CD8 (CD8+ Tcells) | HPA037756 | Sigma Aldrich |
| PD-1 (Exhaustion) | HPA035981 | Sigma Aldrich |
| M2 macrophages CD163 | HPA046404 | Sigma Aldrich |
| CD4 (CD4+ T cells) | HPA004472 | Sigma Aldrich |
| FOXP3 (regulatory T-cells) | 98377 | CellSignaling |

**Table S5:** Immune related gene signatures.

| Signature | Ref. | Genes |
| --- | --- | --- |
| T-cell exclusion | (3) | *BZW2, CCT3, CDK4, GPATCH4, ISYNA1, MDH2, PPIA, RPL31, RPL37A, RPL41, RPS21, RPS27A, RUVBL2, SAE1, UBA52, AHCY, C19orf48, C1QBP, CCT6A, CHCHD2, CTPS1, EEF1G, EIF2S3, EIF3K, EIF4A1, FARSA, FBL, FKBP4, GAS5, GNB2L1, GPI, HNRNPA1, HNRNPC, IDH2, ILF2, NACA, NCL, NME1, NOLC1, PABPC1, PAICS, PFN1, POLD2, PPA1, PTMA, PUF60, RPL10A, RPL11, RPL12, RPL13, RPL13A, RPL13AP5, RPL17, RPL18, RPL18A, RPL21, RPL26, RPL27, RPL28, RPL29, RPL3, RPL36, RPL36A, RPL37, RPL4, RPL5, RPL6, RPL8, RPLP0, RPLP1, RPS10, RPS11, RPS13, RPS14, RPS15, RPS15A, RPS16, RPS17, RPS17L, RPS18, RPS19, RPS23, RPS24, RPS27, RPS28, RPS3, RPS4X, RPS5, RPS6, RPS7, RPS8, RPS9, RPSA, RRS1, SERPINF1, SET, SHMT2, SLC19A1, SLC25A13, SNHG6, SNRPE, SOX4, SSR2, TIMM50, TOP1MT, TUBB, UQCRFS1, UQCRH, VDAC2, APP, ATP5D, ATP5G3, BOP1, BTF3, C6orf48, CACYBP, CCT4, CCT7, CDCA7, DARS, DCTPP1, DDX21, EEF1B2, EEF1D, EEF2, EIF3E, EIF3F, EIF3G, EIF3M, ENO1, EXOSC5, FAM92A1, GGH, GNL3, HMGB1, HNRNPH1, HNRNPM, HSPD1, IFRD2, ILF3, IMPDH2, LDHB, LSM4, LSM7, LYPLA1, MAGEC1, MCM7, MKI67IP, MRPL15, MRPL37, MRPL4, MRPS12, NDUFA11, NME2, NOP16, NPM1, NREP, PLEKHJ1, POLR1D, POLR2E, PRMT1, RPL10, RPL14, RPL15, RPL19, RPL22, RPL27A, RPL30, RPL32, RPL35, RPL39, RPL7, RPL7A, RPL9, RPLP2, RPS2, RPS20, RPS25, RPS3A, RQCD1, RSL1D1, SERBP1, SLC25A6, SMARCA4, SMIM15, SNHG15, SNRPB, SNRPC, SNRPD1, SNRPD2, SRM, SSB, TIMM13, TIMM44, TPI1, TRAP1, TRIM28, TYMS, UCK2, UHRF1, XIST, ZFAS1* |
| Inflamed | (4) | *IRF1, CD8A, CCL2, CCL3, CCL4, CXCL9, CXCL10, ICOS, GZMK, HLA-DMA, HLA-DMB, HLA-DOA, HLA-DOB* |
| Expand immune | (5) | *CXCR6, CD3D, CD2, ITGAL, TAGAP, CIITA, HLA-DRA, PTRPC, CXCL9, CCL5, NKG7, GZMA, PRF1, CCR5, CD3E, GZMK, IFNG, HLA-E, GZMB, PDCD1, SLAMF6, CXCL13, CXCL10, IDO1, LAG3, STAT1, CXCL11* |
| IFNG | (5) | *IFNG, STAT1, CCR5, CXCL9, CXCL10, CXCL11, IDO1, PRF1, GZMA, HLA-DRA* |
| CTL | (6) | *CD8A, CD8B, GZMA, GZMB, PRF1* |
| CD8 | (6) | *CD8A, CD8B* |
| CYT | (7) | *GZMA, PRF1* |
| CD8 T-cell exhaustion | (8) | *PDCD1, LAYN, HAVCR2, LAG3, CD244, CTLA4, LILRB1, TIGIT, TOX, VSIR, BTLA, ENTPD1, CD160, LAIR1* |
| Core NFkB | MSigDB:M8804 (9) | *BCL3, CHUK, NFKBIA, NFKBIB, NFKBIE, IKBKB, IKBKE, IKBKG, NFKB1, NFKB2, REL, RELA, RELB* |
| IRF3 targets | MSigDB:M5133 (10) | *B4GALT5, IFIT3, ISG15, IFI44, ARG2, PMAIP1, GBP1, F13B, AHNAK, OAS2, NR3C1, IFIT1, PLCG2* |
| STING signaling | MSigDB:M7982 (11) | *ADAR, IFNA8, IFNA1, IFNB1, IRF1, IRF2, IRF6, IRF7, IRF9, CCL5* |
| Interferon α response | MSigDB:M5911 (12) | *PARP12, PARP14, PARP9, PLSCR1, PNPT1, PROCR, PSMA3, PSMB8, PSMB9, PSME1, PSME2, RIPK2, RNF31, RSAD2, RTP4, SAMD9, SAMD9L, SELL, SLC25A28, SP110, STAT2, TAP1, TDRD7, TENT5A, TMEM140, TRAFD1, TRIM14, TRIM21, TRIM25, TRIM26, TRIM5, TXNIP, UBA7, UBE2L6, USP18, WARS1* |

**Table S6:** Results of multivariable survival analysis using Cox regression for BRCAness and estimated high vs low CD8 T cell fraction in the TCGA cohort.CI, confidence interval, Sign., significance level *P*<0.001 ***, *P*<0.01 **, *P*<0.05 *; HR, hazard ratio

| Variable | HR | 95% -CI | p | Sign. |
| --- | --- | --- | --- | --- |
| BRCAness *vs* noBRCAness | 0.51 | (0.35-0.75) | 0.00062 | *** |
| Estimated CD8 T cell fraction high *vs* low | 0.81 | (0.56-1.19) | 0.28 |  |
| Tumor residual disease | 2.99 | (1.59-5.61) | 0.00067 | *** |
| Age | 1.03 | (1.01-1.04) | 0.0025 | ** |
| FIGO Stage 2 vs.3 | 0.97 | (0.30-3.11) | 0.96 |  |
| FIGO Stage 3 vs 4 | 1.50 | (0.44-5.07) | 0.52 |  |

**Table S7:** Lists of selected genes associated with different immune related functions corresponding to Figure 4A.

| Signature | Genes |
| --- | --- |
| Cytotoxic effector function | *CD8A, CD8B, EOMES, FASLG, GNLY, GZMA, GZMB, GZMK, IFNG, PRF1, TBX21, ZAP70* |
| Immune response | *IRF1, CD8A, CCL2, CCL3, CCL4, CXCL9, CXCL10, ICOS, GZMK, HLA-DMA, HLA-DMB, HLA-DOA, HLA-DOB* |
| Antigen processing and presentation | *B2M, CD1C, CD1D, CD74, CD83, CIITA, HLA-A, HLA-B, HLA-C, HLA-DMA, HLA-DMB, HLA-DOA, HLA-DOB, HLA-DPA1, HLA-DPB1, HLA-DQA1, HLA-DQA2, HLA-DQB1, HLA-DQB2, HLA-DRA, HLA-DRB1, HLA-E, HLA-F, HLA-G, MR1, PSMB7, PSMB8, PSMB9, TAP1, TAP2, TAPBP* |
| Immune checkpoints | *ADORA2A, BTLA, BTNL2, CD160, CD2, CD200, CD200R1, CD226, CD244, CD27, CD274, CD276, CD28, CD40, CD40LG, CD47, CD48, CD58, CD69, CD70, CD80, CD86, CD96, CEACAM1, CTLA4, ENTPD1, EOMES, HAVCR1, HAVCR2, HHLA2, ICOS, ICOSLG, IDO1, IDO2, KDR, KIR2DL4, KLRC1, KLRK1, LAG3, LAIR1, LGALS9, LTA, MICA, MICB, NCR3, NCR3LG1, NECTIN2, NECTIN3, NT5E, PDCD1, PDCD1LG2, PVR, RAET1E, SIRPA, SIVA1, SLAMF1, SLAMF6, TDO2, TIGIT, TIMD4, TMEM173, TMIGD2, TNFRSF13B, TNFRSF13C, TNFRSF14, TNFRSF17, TNFRSF18, TNFRSF25, TNFRSF4, TNFRSF8, TNFRSF9, TNFSF13, TNFSF13B, TNFSF14, TNFSF15, TNFSF18, TNFSF4, TNFSF8, TNFSF9, TRAF1, TRAF2, TRAF3, ULBP1, VSIR, VTCN1* |
| INFG | *STAT1, CCR5, CXCL9, CXCL10, CXCL11, IDO1, PRF1, GZMA, HLA-DRA* |
| T-cell exhaustion | *HAVCR2, PDCD1, ENTPD1, TNFRSF9, SIRPG, CTLA4, CXCL13, TOX, MYO7A, VCAM1, TIGIT, LAG3, NDFIP2, ACP5, DUSP4, MIR155HG, PHLDA1, CCL3, IFNG, GZMB, PARK7, CXCR6, CD27, FKBP1A, BST2, CD63, CD27-AS1, ITGAE, HLA-DRA, IGFLR1, CTSD, CD38, ITM2A, SNAP47, LAYN, KRT86, TNFRSF18, RBPJ, CD82, RGS1* |
| TGFb/CAF marker | *RCN3, ZCCHC24, BMP4, TNFRSF8, TDO2, INHBA, NTM, FAP, ACTA2, ASPN, COL11A1, CSPG4, DES, FAP, FN1, FOXF1, ITGA11, ITGB1, MFAP5, MME, OGN, P4HA1, P4HB, PDGFRA, PDGFRB, PDPN, POSTN, S100A4, SLC16A4, SPARC, THY1, TNC, VIM, ZEB1* |

# References

1. Takamatsu S, Yoshihara K, Baba T, Shimada M, Yoshida H, Kajiyama H, Oda K, Mandai M, Okamoto A, Enomoto T, et al. Prognostic relevance of HRDness gene expression signature in ovarian high-grade serous carcinoma; JGOG3025-TR2 study. *Br J Cancer* (2023) 128:1095–1104. doi: 10.1038/s41416-022-02122-9

2. Altman D, Lausen B, Sauerbrei W, Schumacher M. Dangers of Using “Optimal” Cutpoints in the Evaluation of Prognostic Factors. *JNCI* (19994) 86:829–835.

3. Jerby-Arnon L, Shah P, Cuoco MS, Rodman C, Su M-J, Melms JC, Leeson R, Kanodia A, Mei S, Lin J-R, et al. A Cancer Cell Program Promotes T Cell Exclusion and Resistance to Checkpoint Blockade. *Cell* (2018) 175:984-997.e24. doi: 10.1016/j.cell.2018.09.006

4. Spranger S, Luke JJ, Bao R, Zha Y, Hernandez KM, Li Y, Gajewski AP, Andrade J, Gajewski TF. Density of immunogenic antigens does not explain the presence or absence of the T-cell–inflamed tumor microenvironment in melanoma. *Proceedings of the National Academy of Sciences* (2016) 113:E7759–E7768. doi: 10.1073/pnas.1609376113

5. Ayers M, Lunceford J, Nebozhyn M, Murphy E, Loboda A, Kaufman DR, Albright A, Cheng JD, Kang SP, Shankaran V, et al. IFN-**γ**–related mRNA profile predicts clinical response to PD-1 blockade. *J Clin Invest* (2017) 127:2930–2940. doi: 10.1172/JCI91190

6. Jiang P, Gu S, Pan D, Fu J, Sahu A, Hu X, Li Z, Traugh N, Bu X, Li B, et al. Signatures of T cell dysfunction and exclusion predict cancer immunotherapy response. *Nat Med* (2018) 24:1550–1558. doi: 10.1038/s41591-018-0136-1

7. Rooney MS, Shukla SA, Wu CJ, Getz G, Hacohen N. Molecular and Genetic Properties of Tumors Associated with Local Immune Cytolytic Activity. *Cell* (2015) 160:48–61. doi: 10.1016/j.cell.2014.12.033

8. Zheng L, Qin S, Si W, Wang A, Xing B, Gao R, Ren X, Wang L, Wu X, Zhang J, et al. Pan-cancer single-cell landscape of tumor-infiltrating T cells. *Science* (2021) 374:abe6474. doi: 10.1126/science.abe6474

9. Gilmore TD. Introduction to NF-kappaB: players, pathways, perspectives. *Oncogene* (2006) 25:6680–6684. doi: 10.1038/sj.onc.1209954

10. Grandvaux N, Servant MJ, tenOever B, Sen GC, Balachandran S, Barber GN, Lin R, Hiscott J. Transcriptional profiling of interferon regulatory factor 3 target genes: direct involvement in the regulation of interferon-stimulated genes. *J Virol* (2002) 76:5532–5539. doi: 10.1128/jvi.76.11.5532-5539.2002

11. Ishikawa H, Barber GN. STING is an endoplasmic reticulum adaptor that facilitates innate immune signalling. *Nature* (2008) 455:674–678. doi: 10.1038/nature07317

12. Liberzon A, Birger C, Thorvaldsdóttir H, Ghandi M, Mesirov JP, Tamayo P. The Molecular Signatures Database (MSigDB) hallmark gene set collection. *Cell Syst* (2015) 1:417–425. doi: 10.1016/j.cels.2015.12.004
